# A multiplexed plant–animal SNP array for selective breeding and species conservation applications

**DOI:** 10.1093/g3journal/jkad170

**Published:** 2023-08-11

**Authors:** Sara Montanari, Cecilia Deng, Emily Koot, Nahla V Bassil, Jason D Zurn, Peter Morrison-Whittle, Margaret L Worthington, Rishi Aryal, Hamid Ashrafi, Julien Pradelles, Maren Wellenreuther, David Chagné

**Affiliations:** The New Zealand Institute for Plant and Food Research Ltd, Motueka 7198, New Zealand; The New Zealand Institute for Plant and Food Research Ltd, Auckland 1025, New Zealand; The New Zealand Institute for Plant and Food Research Ltd, Palmerston North 4410, New Zealand; USDA-ARS National Clonal Germplasm Repository, Corvallis, OR 97333, USA; Department of Plant Pathology, Kansas State University, Manhattan, KS 66506, USA; The New Zealand Institute for Plant and Food Research Ltd, Nelson 7010, New Zealand; University of Arkansas, Fayetteville, AR 72701, USA; Department of Horticultural Science, North Carolina State University, Raleigh, NC 27695, USA; Department of Horticultural Science, North Carolina State University, Raleigh, NC 27695, USA; Labogena, Jouy-en-Josas 78350, France; The New Zealand Institute for Plant and Food Research Ltd, Nelson 7010, New Zealand; School of Biological Sciences, University of Auckland, Auckland 1010, New Zealand; The New Zealand Institute for Plant and Food Research Ltd, Palmerston North 4410, New Zealand

**Keywords:** *Rubus* spp, *Leptospermum scoparium*, *Chrysophrys auratus*, *Pseudocaranx georgianus*, DNA pooling, SNP markers

## Abstract

Reliable and high-throughput genotyping platforms are of immense importance for identifying and dissecting genomic regions controlling important phenotypes, supporting selection processes in breeding programs, and managing wild populations and germplasm collections. Amongst available genotyping tools, single nucleotide polymorphism arrays have been shown to be comparatively easy to use and generate highly accurate genotypic data. Single-species arrays are the most commonly used type so far; however, some multi-species arrays have been developed for closely related species that share single nucleotide polymorphism markers, exploiting inter-species cross-amplification. In this study, the suitability of a multiplexed plant–animal single nucleotide polymorphism array, including both closely and distantly related species, was explored. The performance of the single nucleotide polymorphism array across species for diverse applications, ranging from intra-species diversity assessments to parentage analysis, was assessed. Moreover, the value of genotyping pooled DNA of distantly related species on the single nucleotide polymorphism array as a technique to further reduce costs was evaluated. Single nucleotide polymorphism performance was generally high, and species-specific single nucleotide polymorphisms proved suitable for diverse applications. The multi-species single nucleotide polymorphism array approach reported here could be transferred to other species to achieve cost savings resulting from the increased throughput when several projects use the same array, and the pooling technique adds another highly promising advancement to additionally decrease genotyping costs by half.

## Introduction

The development of medium-density genotyping tools for the inexpensive, rapid, and reliable screening of hundreds of samples is of utmost importance in molecular breeding programs and for the management of wild populations and germplasm collections. Nowadays, there are several high-throughput genotyping technologies and platforms, each with advantages and disadvantages. Whole genome sequencing (WGS) is increasingly being used; however, it can be a costly approach for the routine screening of large numbers of samples, particularly in species with large genome sizes. While reduced-representation genotyping-by-sequencing [GBS (Elshire *et al*. 2011); restriction-site associated DNA sequencing (Davey and Blaxter 2010)] is more scalable, such methods generate high-error rates and missing data (Lowry *et al*. 2017). Additionally, extensive bioinformatics resources are required for the curation of GBS datasets (Bilton *et al*. 2018). Single nucleotide polymorphism (SNP) arrays generate genotype data that are more reliable than GBS (Montanari *et al*. 2019; Vanderzande *et al.* 2020), and their costs can be relatively low, particularly in comparison with WGS approaches, making them an efficient tool for high-throughput genotyping of breeding, as well as mapping, populations. One limitation of SNP arrays is that the identification of variants and the design of efficient SNP probes depend on the availability of a well-assembled low-error rate reference genome. However, this requirement has become less of a problem recently, as high-quality genomes have been developed for many commercially and ecologically relevant species (Hotaling *et al*. 2021; Sun *et al*. 2021). The main disadvantage of SNP arrays is the ascertainment bias caused by uneven representation of diversity in the re-sequencing panels during the polymorphism detection step. However, that is not a major concern if SNP arrays are applied to screen populations that are genetically related to the re-sequencing panels. SNP arrays have proven particularly useful for genome-informed breeding applications, such as checking sample identity, pedigree, and relationship assignment, trait mapping, and genomic predictions (Montanari *et al*. 2020; Muranty *et al*. 2020; Sideli *et al*. 2020; Zurn *et al*. 2020b; Jurcic *et al*. 2021; Zhou *et al*. 2021). Nonetheless, when applying SNP arrays to screen wild/natural populations, increased attention needs to be given to the possibility that genetic variation can be missed. This is particularly likely in cases where the re-sequencing panels used for polymorphism detection are small and/or are not collected across the same spatial scale as subsequently tested samples. Nevertheless, SNP arrays can be powerful tools for wild population analyses, provided the polymorphism detection panels are large and diverse, and they can address questions related to population structure, provenance, and kinship.

A large number of SNP arrays have been designed for plant and animal species important for primary production (e.g. agriculture, horticulture, and aquaculture), with densities ranging from a few hundred to several thousand markers (Chagné *et al*. 2019b; Montanari *et al*. 2019; Saint-Pé *et al*. 2019; You *et al*. 2019; Morales *et al*. 2020; Sun *et al*. 2020; Vanderzande *et al.* 2020; Mastrochirico-Filho *et al*. 2021; Peñaloza *et al*. 2021 to cite some of the most recent). In some cases, researchers have exploited the known synteny among genomes of sister or closely related species and developed SNP arrays that combine markers from different genera. Key examples include the apple (*Malus domestica*) and pear (*Pyrus communis*) Illumina Infinium II 9K SNP array (Montanari *et al*. 2013), the Pacific (*Crassostrea gigas*) and European oysters (*Ostrea edulis*) Axiom 60K SNP array (Gutierrez *et al*. 2017), the “MedFish” Axiom 60K SNP array for European seabass (*Dicentrarchus labrax*) and gilthead seabream (*Sparus aurata*) (Peñaloza *et al*. 2021), and the Axiom SerraSNP array with ∼30K SNPs each for the South American fresh water fishes pacu (*Piaractus mesopotamicus*) and tambaqui (*Colossoma macropomum*) (Mastrochirico-Filho *et al*. 2021). However, multi-species SNP arrays designed for different and completely unrelated taxa with the purpose of pooling DNA from two (or more) samples have not been published thus far. The development and validation of such an array was the objective of this work, in an effort to create a tool for routine, medium-density genotyping of hundreds of samples from breeding programs as well as wild populations for four genera with rapidly growing primary production industries. These included two plant and two fish genera, specifically *Rubus*, *Leptospermum*, *Chrysophrys*, and *Pseudocaranx*.

The *Rubus* genus of the Rosaceae family comprises red (*Rubus idaeus*) and black (*Rubus occidentalis*) raspberries, as well as blackberries (*Rubus* subgenus *Rubus*). Simple sequence repeat markers were used to confirm identity and assess diversity at the Agricultural Research Service of United States Department of Agriculture (USDA-ARS)—National Clonal Germplasm Repository (NCGR) (Dossett *et al*. 2012; Zurn *et al*. 2018), and target capture sequencing was used for phylogenetic analyses (Carter *et al*. 2019). However, while genetic maps and reference genomes are now available for *Rubus* (Ward *et al*. 2013; Bushakra *et al*. 2015; VanBuren *et al*. 2016; Hackett *et al*. 2018; Brůna *et al*. 2022), the application of genomics resources in these crops is still limited (Foster *et al*. 2019). Similarly, the shrub *Leptospermum scoparium*, which includes mānuka from Aotearoa-New Zealand (NZ), is an undomesticated species supporting the production of honeys with unique high antimicrobial properties, which attract premium prices. The recent publication of a reference genome for *L. scoparium* (Thrimawithana *et al*. 2019) and the re-sequencing of specimens from across its natural range (Koot *et al*. 2022) have opened new avenues for the development of genomic resources for this species to support its management and selective breeding. Emerging animal species of economic or ecological importance would also benefit from an increased application of genomic tools. Australasian snapper (*Chrysophrys auratus*, tāmure) and silver trevally (*Pseudocaranx georgianus*, araara) are two candidate species for aquaculture in NZ. Selective breeding programs for both these finfish species have recently been initiated to diversify the aquaculture sector (Ashton *et al*. 2019a, 2019b; Valenza-Troubat *et al*. 2022a). Mānuka, tāmure, and araara are native to NZ and considered treasures (taonga) by Māori (Morgan *et al*. 2019), who have traditional uses for them as sources of food and medicine.

This study describes the design of a multi-species plant–animal 60K SNP array that combines 13K SNP markers for *Rubus*, 9K for mānuka, 18K for snapper, and 20K for trevally, and its validation by the screening of more than 6,000 pooled plant/fish DNA samples. The potential of DNA pooling as a strategy to reduce genotyping costs was also evaluated. Pedigree reconstruction and genetic diversity analyses were performed to demonstrate the use of the SNP array. Finally, cross-amplification in species closely related to snapper (Japanese red seabream, *Chrysophrys*/*Pagrus major*) and silver trevally (yellowtail kingfish, *Seriola lalandi*) was examined.

## Materials and methods

### Sequencing, variant calling, and SNP filtering

Different sequencing datasets were available for each of the four organisms targeted in this study and the variant calling and filtering analyses performed are described in Appendix A (*Rubus* spp.), Appendix B (mānuka), Appendix C (snapper), and Appendix D (trevally).

### SNP final selection and array design

As raspberry variants were generated from GBS performed in bi-parental populations, genotypic data were imported into JoinMap v5.0 (VanOoijen 2006) to identify a subset of “validated SNPs” that successfully grouped into linkage groups (LGs). Additionally, 859 raspberry SNPs designed on candidate genes that control sugar content (Zurn *et al*. 2020a) were added to the dataset.

For the selected SNPs for each of the species, 60 bp up- and down-stream flanking sequences were extracted from the respective reference genomes and submitted to Thermo Fisher Scientific for quality scoring. Only SNPs for which at least one probe was recommended (pconvert > 0.6, no wobbles, and poly count = 0) were kept. BLASTn (v2.6.0) analysis was then performed to remove plant SNPs with flanking sequences that showed high similarity (-evalue 1 × 10^−5^ -perc_identity 0.8) to fish DNA, and *vice versa*, using the reference genomes for all four species, to avoid cross-hybridization between fish and plant DNA. Finally, SNPs that were too close to the start of the chromosome/scaffold (<60 bp), and for which flanking sequences could not be extracted, were eliminated.

For the raspberry SNPs, alignments to a *R. idaeus* “Heritage” draft genome (Driscoll's, Watsonville, CA, USA; unpublished) were checked, and those that had either no hits or more than one mismatch were discarded. This quality check was necessary because the SNPs were designed on the *R. occidentalis* reference genome, the only raspberry genome publicly available at this time, while target populations for application of this SNP array are mainly derived from *R. idaeus*.

Finally, probes for all selected SNPs were tiled on an Applied Biosystems Axiom myDesign array (Thermo Fisher Scientific). A set of 8,000 Dish Quality Control (DQC) probes (200 for each of the four organisms) were also included for quality control at genotyping. These probes were designed from non-polymorphic genome locations, determined by examining the sequences of the individuals used for variant calling.

### DNA extractions

SNP array genotyping reactions were performed by multiplexing one fish and one plant DNA sample in equal quantities, as specified below. *Rubus* and mānuka leaf samples were collected by a consortium of different institutes and DNA extracted in the respective laboratories. *Rubus* DNA extractions were performed in 96-well plates from freeze-dried tissue using commercial kits [e.g. Macherey-Nagel Nucleospin kit was used at Plant & Food Research (PFR)]. Mānuka samples were collected in natural stands in remote locations using silica beads in 2 mL screw cap tubes as described in Koot *et al*. (2022) and DNA was extracted using a modified CTAB protocol (Doyle and Doyle 1987; Chagné *et al*. 2019a). Fish DNA extractions were performed using fin clip samples collected in 96-well plates and using a proprietary automated protocol by Slipstream Automation Ltd (Palmerston North, NZ). All fish and plant DNA samples were then dried for shipping to Labogena (Jouy-en-Josas, France), where they were quantified using fluorometry and normalized to 2 *μ*g. DNA from one fish and one plant sample were then pooled and processed for genotyping.

### SNP array genotyping

Two separate batches of genotyping were performed, amounting to 2,686 and 3,455 samples, respectively, and including both pooled plant/fish DNA samples and single-species ones. Specifically, these were 49 *Rubus* only samples, 511 mānuka only, 214 Australasian snapper only, 322 silver trevally only, 2,908 *Rubus* + snapper pooled samples, 39 *Rubus* + Japanese red seabream, 225 *Rubus* + trevally, 23 *Rubus* + kingfish, 1,122 mānuka + snapper, and 656 mānuka + trevally (Supplementary Table 1). In total, 3,244 *Rubus*, 2,289 mānuka, 4,244 snapper, 1,203 trevally, 39 red seabream, and 23 kingfish samples were genotyped.

The SNP data were automatically split for the four sets of SNPs (*Rubus*, mānuka, snapper, and trevally) and analyzed separately in the Axiom Analysis Suite v5.1.1 software (https://www.thermofisher.com/us/en/home/life-science/microarray-analysis/applications/predictive-genomics/population-genomics/software.html). The data were quality-filtered using a DQC threshold of 0.82 (default) and a QC call rate threshold of 95, and genotypes were called with the default parameters. The *OffTargetVariants* (OTV) caller was run on the OTV, i.e. SNPs that might contain null alleles.

### SNP validation

The effectiveness of the SNP array was evaluated by verifying if the expected population structure could be depicted in subsets of samples for all four organisms. Only the higher quality SNPs [*PolyHighResolution* (PHR) SNPs and *NoMinorHom* (NMH)] were used for these analyses. These are SNPs that show two (NMH) or three (PHR) clear and well-separated clusters for each genotypic class (AA/AB/BB). The methods applied in each species are described in Appendix A (*Rubus* spp.), Appendix B (mānuka), Appendix C (snapper and seabream), and Appendix D (trevally).

### Comparison of genotyping quality among species

The quality parameters DQC, QC call rate, and call rate were compared among species using boxplots. The effect of DNA pooling on the quality of genotyping was also assessed for each of the four main species by generating boxplots, calculating descriptive statistics such as mean, standard deviation (SD), and median, and running an unequal variance (independent) *T*-test (or Welch *T*-test) in the R stats v4.0.0 package. Correlations between quality values of plant and fish samples from the same reaction were depicted in scatter plots. All samples submitted for genotyping were used for these comparisons.

### Ethics statement

Informed consent was granted verbally by Māori landowners to re-use the reference genome of *L. scoparium* “Crimson Glory” (Thrimawithana *et al*. 2019), as well as the pool-sequencing data and DNA samples of Koot *et al*. (2022), for the purpose of developing and evaluating the SNP array in mānuka. The consent was given during a project meeting in June 2020 and minutes were documented. All trevally research carried out in this study was reviewed and approved by the animal ethics committee of Victoria University of Wellington in NZ (application number 25976). For snapper, ethics approval was granted through Victoria University of Wellington in NZ (application number 2014R19) and the University of Auckland (Ref. 002169).

## Results

### Multi-species SNP array design

#### *Rubus* spp.

*Raspberry*. Variants were called from GBS datasets of seven F_1_*Rubus* subgenus *Idaeobatus* populations (Supplementary Table 2). The total numbers of variants called from the populations X14.102, X16.015, and NC493×Chilliwack (CW) datasets were, respectively, 1,335,709, 406,253, and 649,597, which were then reduced in turn to 432,433, 239,830, and 403,211 SNPs after initial basic filtering; 26, 1, and 10 samples, respectively, with high rate of missing data were removed from each population. For the dataset including families X16.093, X16.095, X16.109, and X16.111, a total number of 53,745 variants were called across 358 samples and filtered down to 52,694 SNPs. Merging of VCF files from all seven populations and thinning resulted in 398,596 unique SNPs with no other neighboring polymorphism. Finally, after extraction of the “validated SNPs” [i.e. SNPs that successfully grouped into LGs in JoinMap v5.0 (VanOoijen 2006)] and removal of the A/T and C/G SNPs, this number reduced to 25,910, including the 859 SNPs associated with sugar content (Zurn *et al*. 2020a). Of these, 23,898 SNPs had at least one probe recommended, and the 9,376 of them that aligned well to the *R. idaeus* “Heritage” genome were included in the array.

*Blackberry*. A total of 4,163 SNPs were selected from a WGS dataset of 27 blackberry accessions, including 1,864 SNPs evenly distributed throughout the genome and 2,299 SNPs within genes of interest potentially associated with sweetness (Zurn *et al*. 2020a), thornlessness (Khadgi and Weber 2021), and flowering (Brůna *et al*. 2022). Of these selected SNPs, 3,719 aligned to a unique position in the *R. occidentalis* reference genome and were submitted to Thermo Fisher Scientific for scoring. The 3,347 loci with recommended probes were included in the final array.

#### Mānuka

Quality filtering of the mānuka pooled sequencing datasets from Koot *et al*. (2022) resulted in 2,006,036 SNPs. Eight classes of SNPs were identified based on their minor allele frequency (MAF) values in each or a combination of gene pools identified by Koot *et al*. (2022) [Northern North Island (NNI), the Central and Southern North Island (CSNI), the East Cape North Island (ECNI), and two gene pools in the South Island (SI) representing the North-East (NESI) and South-West of the South Island (SWSI); Supplementary Table 3], and a total of 42,122 SNPs were identified that fit into these classes. Thermo Fisher Scientific scoring classified 33,484 of these SNPs as recommended, and a random set of 9,002 SNPs evenly distributed in the genome was selected for inclusion in the array.

#### Snapper

A total of 6,255,825 SNPs resulted from variant calling on 80 re-sequenced samples from the PFR snapper breeding program, reduced to 4,151,564 after thinning with a 30 bp window. No SNP site exhibited >20% missing data, and filtering for maximum depth (DP), MAF, multi-allelic and A/T and C/G SNPs resulted in a total of 2,419,846 SNPs. After linkage disequilibrium (LD) pruning, 26,719 SNPs were left, of which 13 were removed because they either aligned to the raspberry or mānuka genomes, or because they were too close to the terminal of the scaffold. Finally, 22,238 SNPs were recommended by Thermo Fisher Scientific scoring, and 18,489 randomly selected SNPs were included in the array. It was noted that 13,204 of these SNPs were in coding regions, according to the male and female gene annotation for the *C. auratus* v1.0 reference genome (Catanach *et al*. 2019).

#### Trevally

The initial SNP calling performed by Valenza-Troubat *et al*. (2022a) from WGS reads of 13 trevally samples resulted in a dataset of 17,795,808 SNPs, which were then thinned to 7,969,343 and subsequently reduced to 3,087,247 after filtering for missing data, maximum DP, MAF, multi-allelic and A/T ad C/G SNPs. Afterwards, LD pruning left 26,666 SNPs and 264 had to be removed because of risk of cross-hybridization with plant genomes or because they were too close to a scaffold terminal. Thermo Fisher Scientific scoring recommended 22,076 SNPs, and 20,234 randomly selected SNPs were successfully included in the array.

In summary, the Axiom multi-species plant−animal 60K SNP array includes 60,448 SNPs from five species and four genera: *R. idaeus*, *R.* subgenus *Rubus*, *L. scoparium*, *C. auratus*, and *P. georgianus* (Table 1; Supplementary Table 4). A higher number of SNPs was included for the fish than for the plant species because of their larger genomes (estimated genome sizes for raspberry, blackberry, and mānuka are 250–300 Mbp, and 700–800 Mbp for snapper and trevally).

**Table 1.** Number of SNP markers for each species in the Axiom multi-species 60K SNP array.

Organism	Species	# SNP markers
Raspberry	*Rubus idaeus/occidentalis*	9,376
Blackberry	*Rubus* subgenus *Rubus*	3,347
Total *Rubus* spp.		12,723
Mānuka	*Leptospermum scoparium*	9,002
Snapper	*Chrysophrys auratus*	18,489
Trevally	*Pseudocaranx georgianus*	20,234
Total		60,448

### Genotyping of test sample sets for each species and validation of the SNP array

#### *Rubus* spp.

**Fig. 1.**
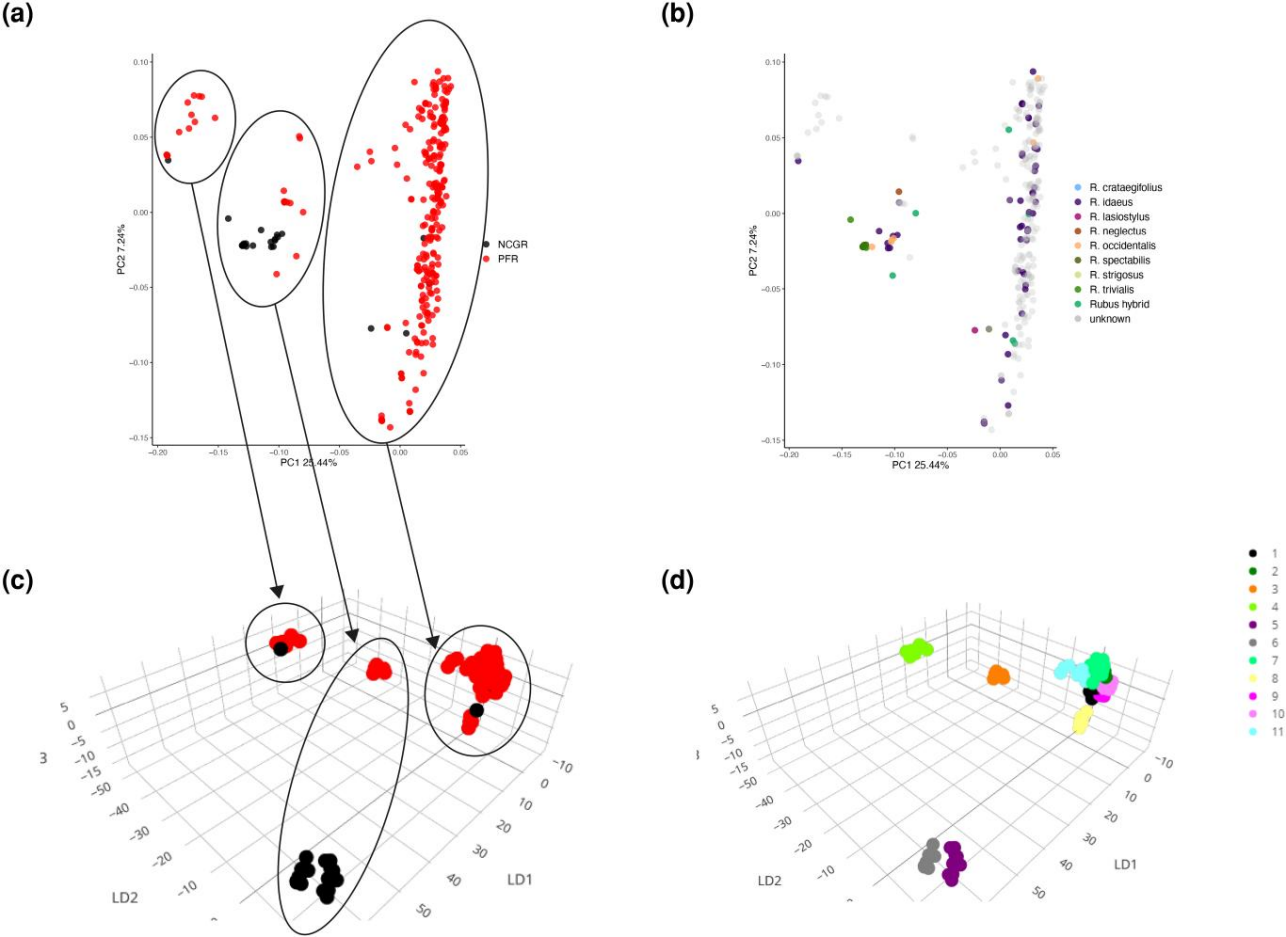
Genetic diversity of diploid *Rubus* samples. PCA with samples colored by repository a) and assigned species b). DAPC plot with samples colored according to repository c) and DAPC clustering d). Circles and arrows in plots a and c show correspondence between PCA and DAPC clustering. NCGR = USDA-ARS National Clonal Germplasm Repository; PFR = The New Zealand Institute for Plant and Food Research Ltd.

*Diploids*. Of the 477 diploid samples analyzed in this study, 57 and 82 failed to pass the DQC and the QC call rate thresholds, respectively, leaving a set of 338 samples for further statistical analyses. Cluster plot assessment highlighted a subset of samples that frequently fell out of the posterior cluster margins (Supplementary Fig. 1), causing errors in the automatic quality evaluation and classification of the SNP markers. A threshold of 0.85 for “allele_deviation_mean” was then established to remove the low-quality samples, leaving 305 samples to be re-analyzed. Finally, 276 samples from PFR germplasm and 29 from the NCGR passed all quality thresholds and exhibited an average call rate of 99.0%. Genotyping resulted in 6,141 SNPs classified as PHR, 1,211 as NMH, 612 as OTV, 1,669 as *MonoHighResolution*, 2,622 as *Other*, and 468 as *CallRateBelowThreshold* (Supplementary Table 5). Two or more replicates were successfully genotyped for four different accessions, including BC 64-9-81 (two replicates), “Glen Ample” (two replicates), “Wakefield” (five replicates), and *Rubus spectabilis* “Gibbs Lake” (three replicates). All replicates had identity-by-state (IBS) >0.97 except for “Wakefield”, which grouped into two sets of duplicated samples each with IBS >0.97. Two of the “Wakefield” samples that were identical to each other but different from the other three were later ascertained to be sampling errors. However, since they were technical replicates from the same sample, they were included in the analysis. Over all the PHR and NMH SNPs verified, 6,885 (93.7%) had no genotyping inconsistencies and were considered robust. Of these, 6,235 were raspberry SNPs and 650 blackberry SNPs. Principal component analysis (PCA) showed three main clusters along the PC1 (25.44% of variation explained), with almost all samples from the NCGR grouping together into one cluster (Fig. 1a). However, there was no correspondence between the PCA clustering and the species assignment (Fig. 1b). A discriminant analysis of principal component (DAPC) was run using the optimal number of 11 clusters, 100 PCs, and 4 DAs. A three-dimensional scatter plot showed a large group that included 7 of the 11 clusters, and four small well-separated groups (Figs. 1c and d). There was good correspondence between the three main clusters identified with the PCA and the three-dimensional separation observed with the DAPC (Fig. 1c). However, the DAPC clusters still could not be explained by the taxonomy of the samples (Fig. 1d).

**Fig. 2.**
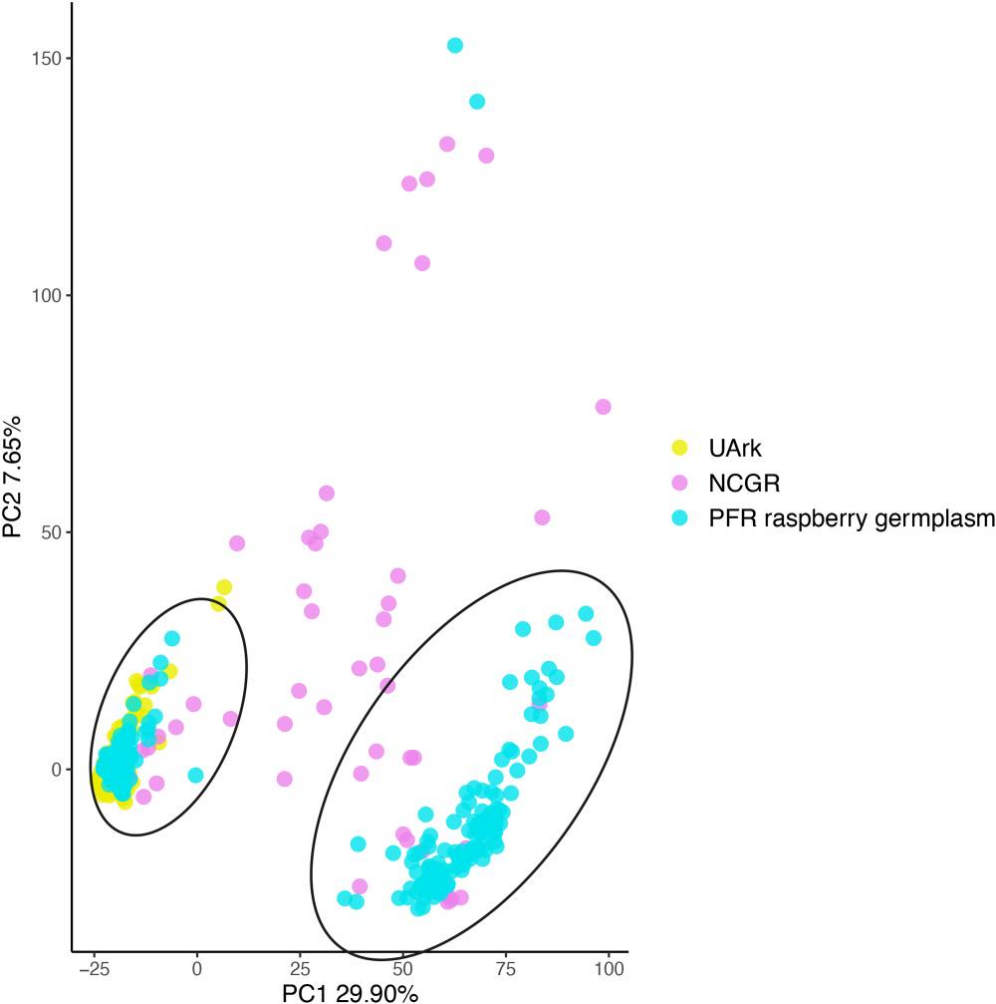
Genetic diversity of tetraploid *Rubus* samples. PCA with samples colored by repository. UArk = University of Arkansas System Division of Agriculture; NCGR = USDA-ARS National Clonal Germplasm Repository; PFR = The New Zealand Institute for Plant and Food Research Ltd.

*Tetraploids*. Tetraploid models were successfully fitted in fitPoly v3.0.0 (Voorrips *et al*. 2011; Zych *et al*. 2019) for 4,872 SNP markers, out of the total 12,723, in 739 samples. This dataset was further reduced to 666 samples and 4,388 SNPs after filtering for missing rate (34% of total *Rubus* SNPs on the array). These included 2,899 raspberry and 1,489 blackberry SNPs. Two main clusters could be observed on the PC1 (29.90% of variation explained) vs PC2 (7.65%) plot, with several samples remaining ungrouped (Fig. 2). As for the diploid samples, the results of the PCA could not be explained by the reported taxonomy; however, there was good correspondence with the repository of origin. Most of the PFR samples were split into the two main clusters, one group overlapping with the University of Arkansas System Division of Agriculture (UArk) samples; ungrouped samples were mostly from NCGR. Samples from PFR and UArk included parental and seedling selections from their breeding programs, while accessions from NCGR represented a diverse range of species and genotypes maintained at their repository for conservation purposes.

#### Mānuka

**Fig. 3.**
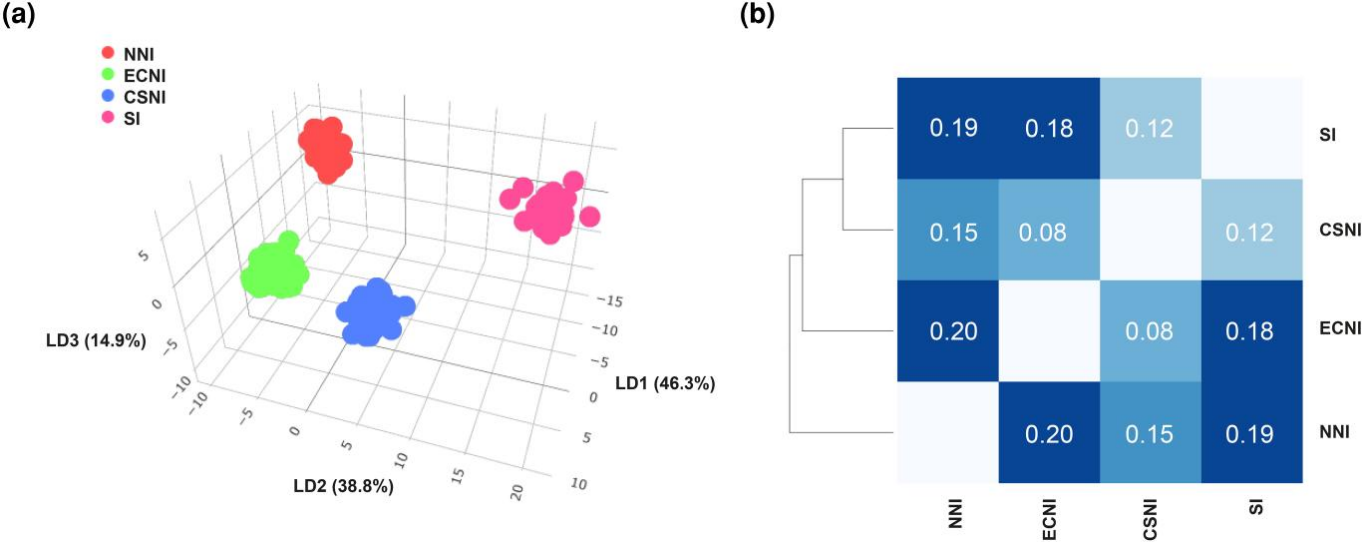
Population genetics analysis of mānuka samples from Aotearoa-NZ. a) DAPC plot with samples colored according to provenance. b) *F_ST_* analysis. NNI: Northern North Island; Central and Southern North Island (CSNI); East Cape North Island (ECNI); SI = South Island.

Of the 264 mānuka samples screened using the array, 233 passed the QC filters. Of the 31 failed samples, 7 were because of a low DQC score and 24 were because of a QC call rate <95. Of the 9,002 SNPs included in the array, 4,969 were classified as PHR, 1,026 as OTV, and 124 as NMH. These 6,119 polymorphic good quality markers represented 68% of the mānuka SNPs included in the array. Of the 2,883 unsuccessful SNPs, 19 were monomorphic (*MonoHighResolution*), 761 had call rate below the threshold, and the majority (2,103) had poor clustering (Supplementary Table 5). When K-means clustering and DAPC analyses were performed using only the 4,969 PHR SNPs, the 233 samples separated into four clusters matching four geographical regions: NNI, CSNI, ECNI, and SI (which included the two pools NESI and SWSI) (Fig. 3a; Supplementary Table 6). *F_ST_* calculated between each of the four regions ranged from 0.08 between ECNI and CSNI, to 0.20 between ECNI and NNI (Fig. 3b).

#### Snapper and seabream

**Fig. 4.**
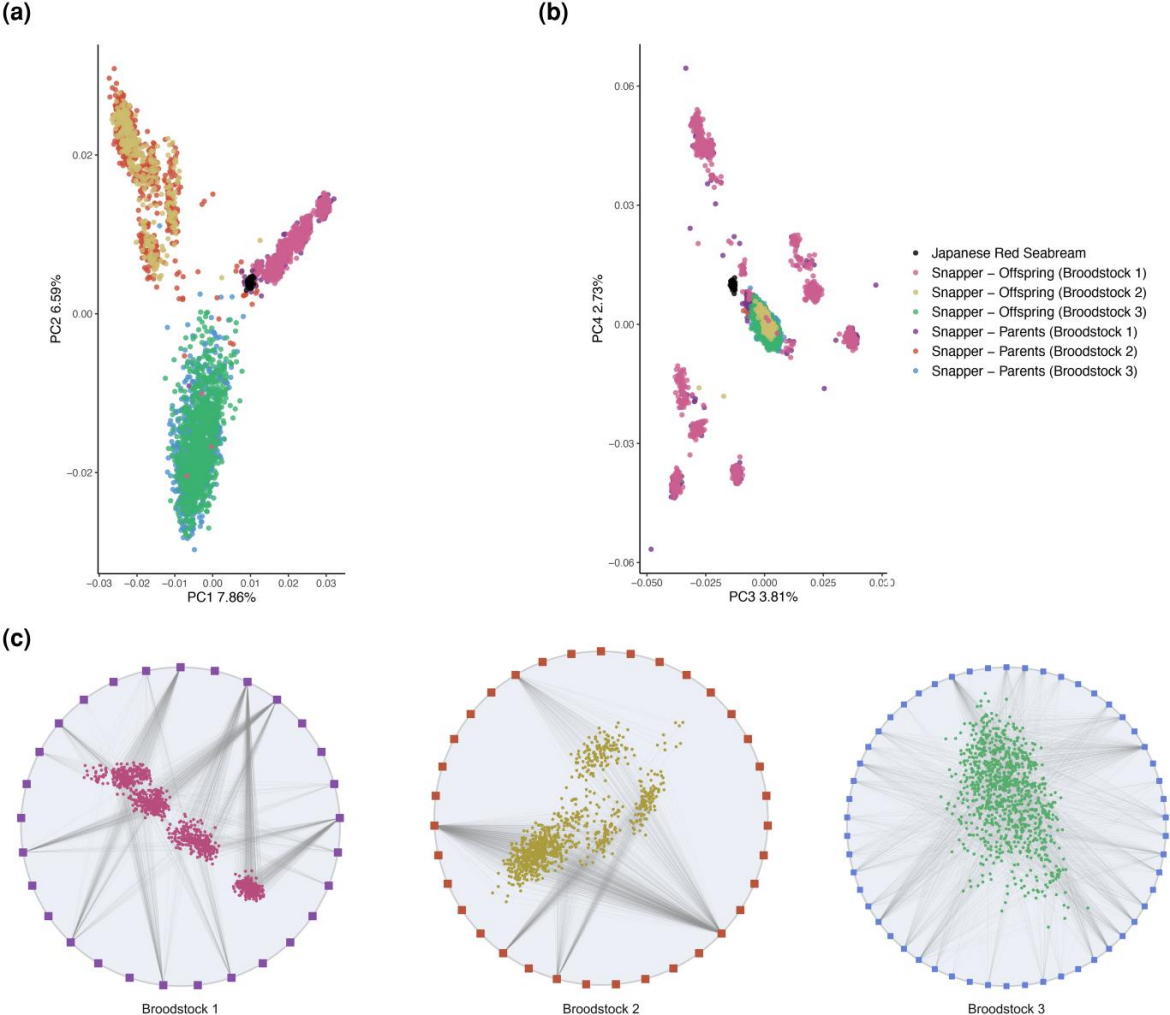
Population genetic analysis and pedigree reconstruction of Australasian snapper and Japanese red seabream. PCA colored by species and population: a) PC1 vs PC2 and b) PC3 vs PC4. c) Pedigree networks: parent-offspring relationships are indicated by a line connecting the adult snapper (square points) and juvenile snapper (circular points) of the three broodstock lines.

Of the 4,244 snapper samples screened using the array, 3,915 passed the QC filters. In the first batch, 48 samples failed because of a low DQC score and 121 because of a QC call rate <95. In the second batch, 23 and 137 samples failed at DQC and QC call rate, respectively. The seabream samples were analyzed together with the snapper samples from the first batch and only 4 out of 39 failed because of low QC call rate. Of the 18,489 SNPs included in the array, in the first batch 11,921 (64.5%) were classified as PHR and 941 (5.1%) as NMH, while the others were monomorphic, OTV, or of poor quality. In the second batch, numbers were similar, with 11,601 (62.8%) PHR and 1,000 (5.4%) NMH. A total of 10,692 and 472 SNPs were classified as PHR and NMH in both batches, respectively (Supplementary Table 5), and were used for subsequent analysis. The variation explained by PC1 and PC2 was 7.86% and 6.59%, respectively. Three major clusters could be observed, corresponding to the snapper broodstock of origin (Fig. 4a). Examination of the PC3 vs PC4 plot (explaining variation of 3.81% and 2.73%, respectively) revealed a major central cluster including broodstocks 2 and 3, while snapper samples from broodstock 1 formed several separate clusters around it (Fig. 4b). The seabream samples overlapped with the snapper broodstock 1 in the PC1 vs PC2 plot, while they formed a distinct cluster in the PC3 vs PC4 plot (Fig. 4b). Broodstock 1 included adult snapper individuals harvested from the wild and their direct offspring spawned in captivity, while broodstocks 2 and 3 included adult individuals generated through PFR's selective breeding program as well as their direct offspring, which were also spawned in captivity. In total, 2,937 trios were detected and confirmed by Mendel test, which resulted in 90.9% of all offspring having assigned parentage (Fig. 4c). Differences were observed among broodstock lines, with 98.3%, 95.5%, and 79.8% of offspring assigned in broodstocks 1, 2, and 3, respectively. In addition, the proportion of adults that contributed to the next generation differed substantially among broodstocks—with 51.7%, 14.3%, and 70.4% of adult fish contributing to offspring generation in broodstocks 1, 2, and 3 respectively (Fig. 4c).

#### Trevally and kingfish

**Fig. 5.**
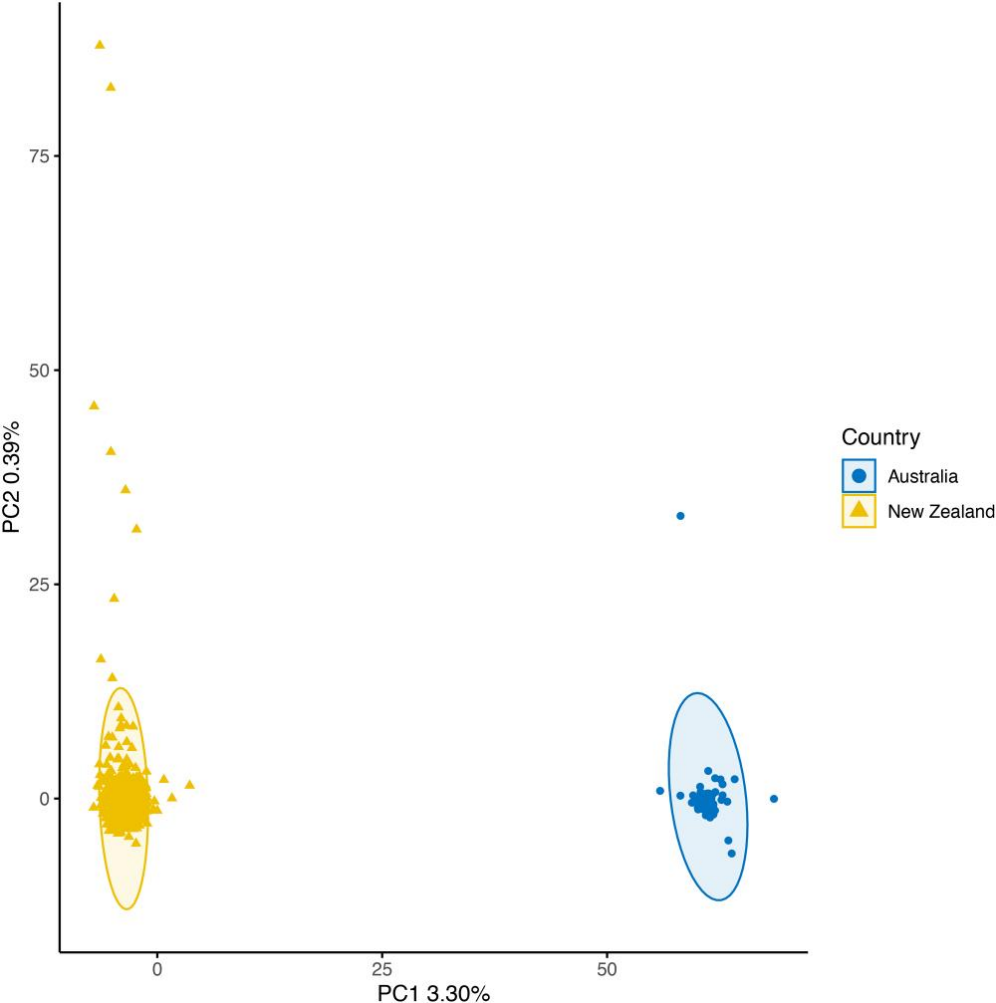
PCA of trevally samples caught in Australia and Aotearoa-NZ.

Since only 48 samples were submitted in the first batch, genotypes for these were called together with all trevally samples from the second batch, and 1,072 out of 1,203 passed all QC filters. Only 10 samples failed the DQC filter, and 121 did not pass the QC call rate threshold of 95. All kingfish samples, which were analyzed together with the trevally, failed at DQC. Of the 20,234 SNPs included in the array, 10,157 were classified as PHR, 1,556 as NMH, 2,521 as OTV, 146 were monomorphic, 1,100 had call rate below the threshold, and 4,754 exhibited poor clustering (Supplementary Table 5). PHR and NMH SNPs were used for subsequent analysis. Of the 978 samples examined for SNP validation, 938 passed the Axiom QC genotyping filters; these included 54 individuals from Australia and 884 from NZ (Supplementary Table 7). The PCA showed a clear separation between Australian and NZ samples along the PC1, which accounted for 3.30% of the variation (Fig. 5). *F_ST_* between the two populations was estimated to be 0.19.

### Evaluation of quality of genotyping

**Fig. 6.**
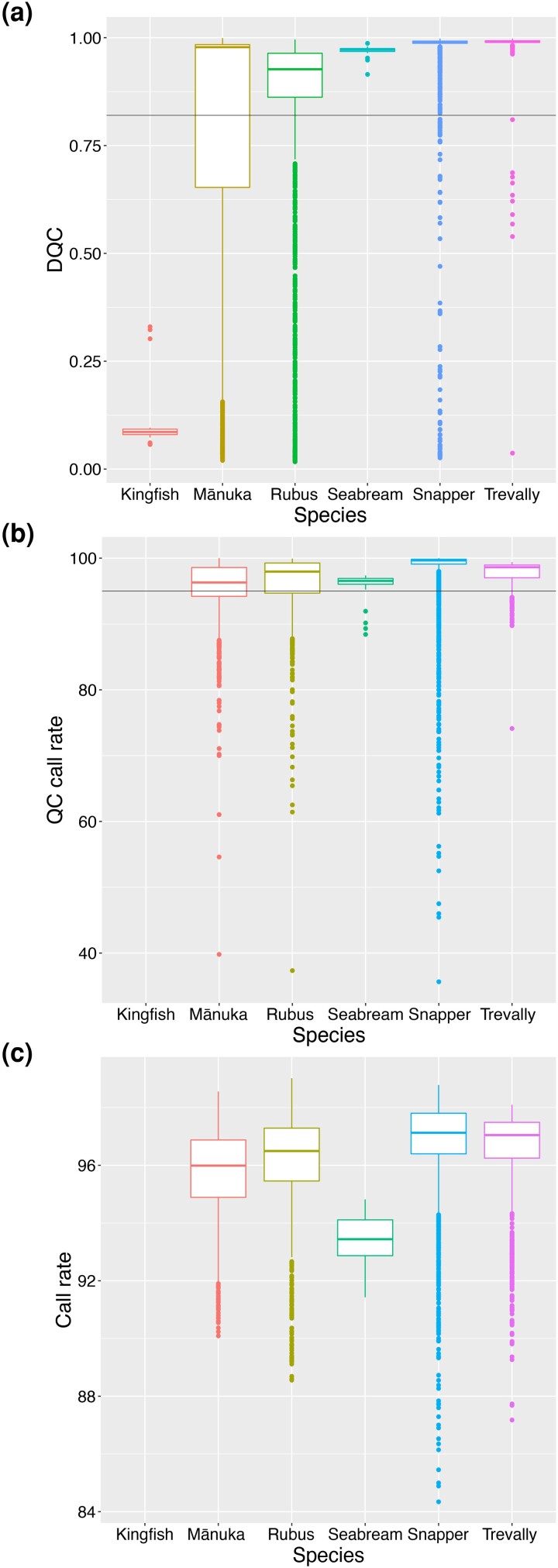
Comparison of genotyping quality parameters among species. Boxplots for DQC a), QC call rate b), and call rate values c) grouped by species.

**Fig. 7.**
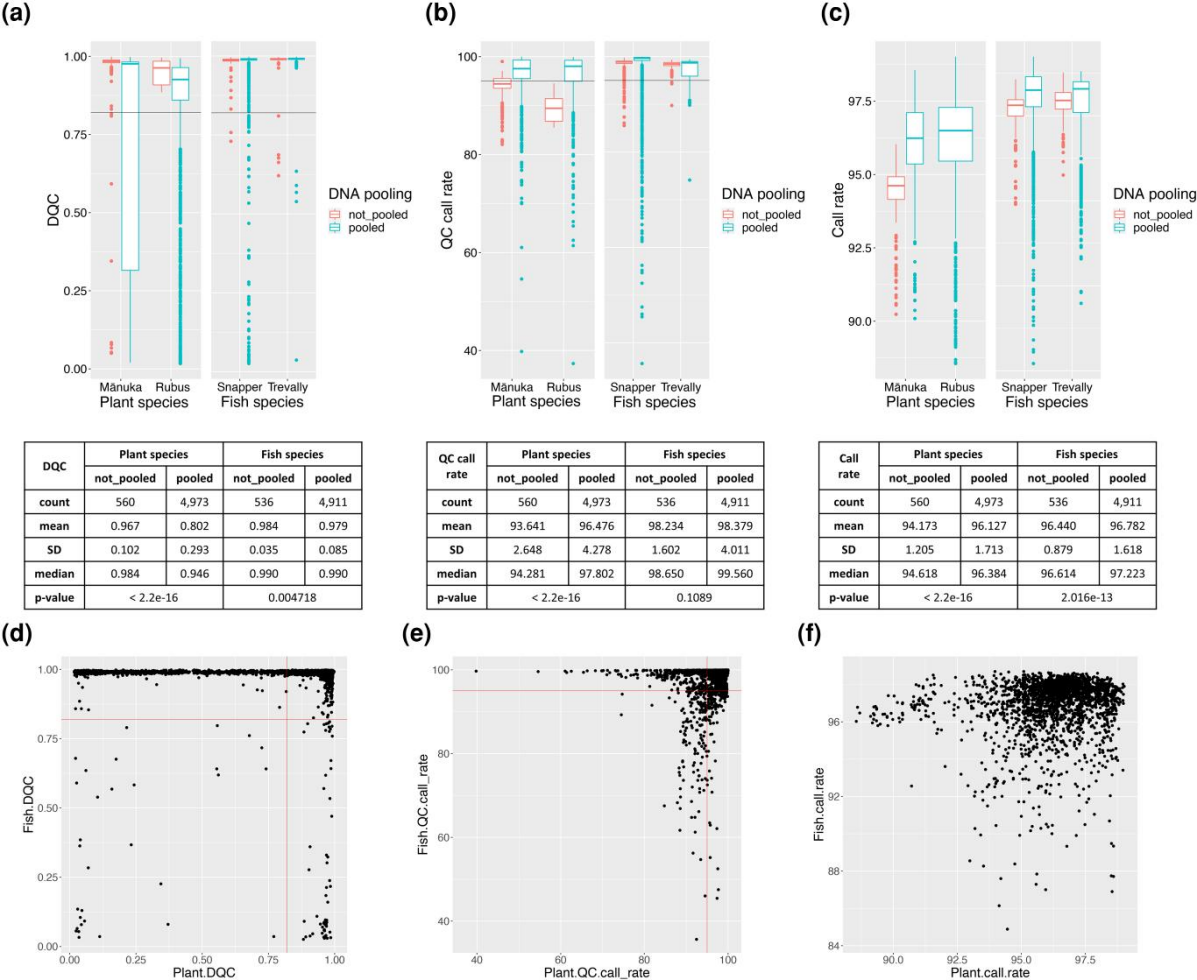
Comparison of genotyping quality parameters between pooled and non-pooled DNA samples. Boxplots for DQC a), QC call rate, b) and call rate values c) grouped by species and pooling, with corresponding values for sample count, mean, SDs, and median, and the T-test *P*-value. Scatter plots for DQC d), QC call rate e), and call rate values f) of pooled plant vs fish samples.

DQC values were always higher in fish samples than in plants, with the exception of kingfish; however, a high variability was observed for all species (Fig. 6a). QC call rate was fairly uniform across all organisms (Fig. 6b), as was call rate for the four main species (Fig. 6c). In both plants and fishes, pooling appeared to have a significant negative effect on the DQC (*T*-test, *P*-value < 0.05); however, differences were more marked in the plant than in the fish samples (Fig. 7a). QC call rate was higher in the pooled plant samples than in the non-pooled ones, but SD was also larger, while the difference was not significant in fish (Fig. 7b). Call rate was higher in pooled than in non-pooled samples in both plants and fish (although none of the *Rubus* non-pooled samples passed QC, thus call rate could not be evaluated) (Fig. 7c). Finally, a large number of multiplexed reactions that returned high DQC values for the fish samples exhibited low DQC for the corresponding plant ones (Fig. 7d), while QC call rate and call rate values were overall in greater agreement between the two (Figs. 7e and f).

## Discussion

Our work demonstrates the successful development of a multi-species plant–animal SNP array and the application of this array in selective breeding programs and the management of natural populations and plant germplasm.

### Novel SNP array developed for two plant and two fish species

This is not only the first published multi-species plant–animal SNP array but also the first array for each of the included organisms *Rubus* spp., mānuka, snapper, and trevally. When looking at the numbers of high-quality SNPs (PHR and NMH), this array had conversion rates of 58%, 56%, 61%, and 58% for each genus, respectively (Supplementary Table 5). These proportions are similar or higher than those of other plant Axiom SNP arrays designed (Bianco *et al*. 2016; Marrano *et al*. 2018; Roorkiwal *et al*. 2018; You *et al*. 2019; Howe *et al*. 2020), and similar or slightly lower than for other aquaculture species (Gutierrez *et al*. 2017; Mastrochirico-Filho *et al*. 2021). The new SNP array developed in this study is a necessary genotyping tool for these increasingly important species, for which researchers have so far had to rely on limited genetic resources.

#### *Rubus* spp.

High-density genotyping in raspberry and blackberry has been carried out mainly via GBS so far (Ward *et al*. 2013; Weber 2014; Bushakra *et al*. 2015; Hackett *et al*. 2018; Jibran *et al*. 2019; Brůna *et al*. 2022), and the development of genetic maps and QTL identification studies have been hampered by the lack of appropriate genetic resources in these species (Foster *et al*. 2019). A more reliable target capture approach was used for a phylogenetic study in *Rubus* (Carter *et al*. 2019); however, this method is not adapted for high-throughput genotyping and only 94 accessions were screened. The SNP array developed here, with its ∼7,000 robust SNPs validated in a diploid dataset, provides a useful tool for the routine genotyping of a large number of samples, necessary for the characterization of germplasm collections and parentage analysis in breeding programs. Additionally, the 6,885 robust SNPs are sufficient for the construction of genetic maps and for QTL mapping analysis, and probably represent an improvement from the high-error and high-missing rate of GBS datasets. This SNP array was also tested on tetraploid *Rubus* spp. samples, and dosage genotypes could successfully be called for 4,388 out of the 12,723 markers. The structure identified in the PCA for the tetraploid samples was well explained by the repository of origin (Fig. 2), suggesting that the genotype calling in this subset of SNPs was correct.

#### Mānuka

Koot *et al*. (2022) used pooled genome re-sequencing to study the genetic structure of mānuka collected across NZ. The SNP array data produced here for a subset of the populations described in Koot *et al*. (2022) agree with the expected clustering whereby NNI, ECNI, and CSNI group separately. However, the two SI provenances SWSI and NESI identified by Koot *et al*. (2022) clustered together in this study (SI), and this is likely to be a result of the bias within the SNP filtering parameters, with more SNPs identifying samples from the SI than from their individual provenances. Where comparable, the *F_ST_* values obtained by both approaches were similar. These findings indicate that the SNP array can be used for reproducible genotyping across mānuka provenances. As for *Rubus* spp., medium-throughput genotyping of mānuka was previously achieved using GBS (Chagné *et al*. 2019a), which required extensive data curation (Bilton *et al*. 2018), while the data generated with the SNP array could be more easily employed in genetic diversity analyses.

#### Snapper

The SNP array was able to discriminate among the different breeding populations of the snapper aquacultured strains, even though these samples were characterized by an overall high degree of relatedness, which reduced the degree of intra-specific genetic diversity. Nonetheless, the SNP data clustered the breeding populations as expected, mirroring the known relatedness among the sampled individuals (Figs. 4a and b). Importantly, the SNP array performed well when used to reconstruct the pedigree of broodstock individuals, with >90% of the offspring successfully assigned to a parental duo. The remaining offspring that could not be assigned to specific parents might derive from individuals that were not genotyped (e.g. because they died before sample collection or because they failed at genotyping). Assignment tests of parents in the broodstock elite lines to offspring indicated skewed parental contributions to the next generation (Fig. 4c), suggesting differing rates of spawning or egg generation, which is supported by previous work applying GBS data (Ashton *et al*. 2019a). Taken together, these results support the accuracy of the SNP markers in snapper and their usefulness for genomic analysis in selective breeding programs.

#### Trevally

The SNP array data showed a clear genetic separation between the trevally sampled in Australia and those caught in NZ waters (Fig. 5). This pattern of wide ranging panmixia is expected for many marine species with high population sizes and high dispersal (Nielsen *et al*. 2009), and has been documented for other marine teleost species in NZ previously [e.g. (Papa *et al*. 2020, 2022; Koot *et al*. 2021)]. Interestingly, the *F_ST_* values between the two clusters were unexpectedly high, denoting pronounced genetic divergence between the two clusters. This could indicate that trevally in NZ and Australia have been geographically isolated for prolonged periods of time and evolved rapidly during this time, possibly because of adaptive pressures to cope with different environmental gradients. Another scenario is that the Australian fishes have been misidentified and they belong to another described or even unknown carangid species in that region. As for the other species reported in this array, only GBS could be used for high-throughput genotyping in trevally so far (Valenza-Troubat *et al*. 2022a, 2022b). This SNP array is easier to use and yields more accurate data than was previously obtained with GBS, hence it represents a step forward in the application of genomics for selection and conservation of this promising aquaculture species.

### Immediate applications of the SNP array data

The SNP validation analyses carried out in this study showed the necessity of accurate genomics tools for evaluating genetic diversity and reconstructing pedigrees for applications in both breeding and conservation practices. For each of the species included in the SNP array, this study highlighted the potential of this tool in solving some of the issues encountered, as well as answering some new research questions.

#### *Rubus* spp.

The *Rubus* data analysis presented some challenges linked to incorrect historical records of ploidy and taxonomy, which this new SNP array might help clarify. *Rubus* species have been reported with ploidies ranging from 2 × to 12×, and the presence of aneuploids (Thompson 1995; Meng and Finn 2002; Hummer *et al*. 2016). Hence, as a first step, samples needed to be separated by ploidy levels to be appropriately analyzed. For most of the samples evaluated here, ploidy was estimated from their assumed parentage. However, given the ease of hybridization among *Rubus* species, even between individuals with different ploidy levels, these estimates have a high-error rate. Flow cytometry is often used to confirm ploidy; however, unusual cytotypes have been observed in *Rubus* at the NCGR (Hummer *et al*. 2016), in agreement with the known occurrence of aneuploidy. Therefore, only a subset of samples from PFR and NCGR that were known to be diploid and tetraploid were analyzed here. Although only robust *Rubus* SNPs were used for PCA and DAPC analyses, clustering did not match the taxonomy of the samples. This may indicate that some samples were not true-to-type, and/or that their taxonomic identification was not accurate. *Rubus* plants easily self-propagate through underground stolons, which can travel far from the mother plant, making their management in the orchard very difficult. Indeed, several genotypes planted next to each other in the PFR orchard proved to be identical. Furthermore, phylogenetic analysis in *Rubus* is challenging because of the common wide inter-species hybridization, apomixis, varying ploidy levels, and remarkable morphologic diversity (Hytönen *et al*. 2018). Currently, more than 500 species are estimated to belong to this genus. Hence, many accessions at the PFR and NCGR germplasm collections have probably been misclassified taxonomically. This SNP array could be extremely helpful in revealing and/or verifying the identity of *Rubus* accessions conserved at PFR and the NCGR, as well as their degrees of relatedness, and in some cases it could even assist in their taxonomic re-classification, as it was shown in a similar study in *Pyrus* (Montanari *et al*. 2020). These are essential analyses for an efficient conservation program, as well as for parental selection for cultivar improvement.

#### Mānuka

This new SNP array is a fundamental tool for a more efficient management and breeding of mānuka populations. *L. scoparium* is a species of ecological, economic, and cultural importance in NZ, where it is considered taonga (treasure) by the indigenous Māori people (Morgan *et al*. 2019). Population genetic studies have already revealed a strong geographically dependent structure in mānuka in NZ (Koot *et al*. 2022), and further insights into its genetic diversity could enable an understanding of its adaptability to different environments, as well as assist in management practices. The possibility to perform genetic screening of mānuka populations easily and effectively will also help in the identification of loci linked to key traits for both mānuka conservation and breeding purposes. Relevant examples are resistance to the tree-killing myrtle rust disease (Smith *et al*. 2020), and increased content of health-beneficial compounds in the nectar that are linked to the production of high-value honey (Van Eaton 2014).

#### Snapper

In snapper and many other bream species used in aquaculture (e.g. gilthead seabream and red seabream), reproduction occurs via mass spawning of selected broodstock lines, and subsequently collected fertilized eggs and reared for on-growing. This means that parental relationships are unknown in these species; hence, genetic tools are necessary for offspring parental assignment. Additionally, high-throughput genotyping with this SNP array will enable quantitative genetic studies, QTL mapping, as well as the estimate of breeding values and inbreeding rates. Routine genetic screening of new wild broodstock lines introduced in captivity, as well as the offspring generated, would also help maintain high genetic diversity in the breeding program and maximize genetic gain.

#### Trevally

The SNP array data generated in this study allowed the discrimination of two different wild trevally populations from Australia and NZ. Even though further work (e.g. mtDNA sequencing) is necessary to confidently resolve whether these represent two distantly related trevally populations or that a different species was instead caught in Australia, this finding indicates that the SNP array can assist in both wild population management and fishery assessments. Furthermore, the application of the SNP array to inform selective breeding of trevally (Valenza-Troubat *et al*. 2022a, 2022b), as demonstrated for snapper, holds immense future potential, e.g. to infer the relatedness of broodstock lines and to inform the selection of suitable outbred parents for new lines.

### The importance of sample quality for successful SNP array genotyping

The Axiom Analysis Suite software provides two parameters to assess sample quality: the DQC, which is based on intensities of non-polymorphic probes (i.e. that do not vary in sequence from one individual to the next) and is expected to be close to 1 for high-quality samples; and the QC call rate, which is the genotyping call rate across a subset of arrayed SNPs selected by Thermo Fisher Scientific. With no previous indication about the quality of the arrayed SNPs, as is normal in new designs like in this study, the QC call rate was not very reliable in determining sample quality. Therefore, we also looked at the call rate over all the SNPs; however, it should be noted that this value is only returned for the samples that are successfully genotyped, i.e. those samples that already passed QC and are therefore considered of higher quality. When looking at the DQC, differences in sample quality were observed between plant and fish samples. A greater failure rate was reported in both *Rubus* and mānuka than in snapper and trevally, which had higher and more uniform DQC values (Fig. 6a). It is important to note, however, that the fish individuals used to design the DQC probes are related to most of the snapper and trevally samples genotyped in this study, making the DQC a very reliable parameter to assess sample quality. On the other hand, it is possible that the DQC probes for *Rubus* and mānuka were not always truly monomorphic, since some of the individuals genotyped are distantly related to those used for the probe design. In terms of QC call rate, there were no evident species-specific differences observed (Fig. 6b), which is consistent with the lower reliability of this parameter in this case. Similarly, call rate values were not too dissimilar among the four main species (only seabream samples showed visibly lower call rates, which is expected, as is discussed below) (Fig. 6c); however, in diploid *Rubus* and mānuka, a number of low-quality samples were identified that fell between genotype clusters and caused errors in the genotypic calls (Supplementary Fig. 1). A similar behavior was not observed in snapper and trevally. Overall, these results suggest that the plant samples had lower quality than the fish. All DNA samples were quantified and normalized; however, snapper and trevally have genomes 2–3 × larger than the diploid *Rubus* and mānuka ones. Additionally, it is possible that differences in the genotyping success rate were caused by DNA quality. Although the quality of the DNA samples was not evaluated, for practical reasons, it is well known that DNA extraction from perennial plants is difficult, particularly because of the presence of polysaccharides and secondary metabolites (Sharma *et al*. 2002; Shepherd and McLay 2011). Often DNA extraction protocols need to be optimized for each species, and scalability to large numbers of samples is particularly difficult to implement. In *Rubus*, for example, CTAB-based extractions [e.g. the Kobayashi method (Kobayashi 1998) or the protocol reported by Porebski *et al*. 1997] are usually recommended over commercial kits; however, these are difficult to implement for high-throughput 96-well plate-based extractions. In mānuka, for samples collected in remote locations, which required leaf tissues to be kept at room temperature and possibly caused DNA degradation, DQC and call rate values were comparatively lower. In future studies, proper DNA quality checking will be necessary to determine if samples are suitable for SNP array genotyping, and optimization of large-scale DNA extraction protocols for recalcitrant species, such as the perennial plants *Rubus* and mānuka, might be important for a higher success rate.

### Multi-species plant–animal SNP array can be run on multiplexed DNA

To the best of our knowledge, this is the first SNP array that exploits the pooling of DNA from different species into a single reaction. Here, samples from two highly divergent orders (teleost fish and dicotyledonous plants) were combined, and genotyping was successfully executed for all the species screened. Several samples were genotyped from non-pooled reactions, allowing us to assess the effect of DNA pooling on the subsequent quality of the results. Overall, samples that were not pooled had a higher success rate than those that came from a mixed reaction; however, differences were observed between fish and plant samples. In snapper and trevally, the differences in DQC between pooled and non-pooled samples were significant but not as marked as in mānuka and *Rubus*, where a larger variability was also observed (Fig. 7a). Interestingly there was a poor correlation between the DQC of fish and plant samples from the same reaction (Fig. 7d). Pooled samples for both plant and fish species showed significantly higher QC call rate and call rate values than non-pooled ones (Figs. 7b and c); although this result seems counterintuitive, it is important to note that a much larger number of pooled samples was evaluated than non-multiplexed ones, which may have biased this comparison. Together these results suggest that the low-quality genotyping in some samples was more likely to be caused by species-specific factors than by the pooling itself. However, it is also possible that the pooling may have disproportionally affected plant DNA because of lower DNA quality compared with the fish DNA. As the genotyping of pooled fish samples was still highly successful, our results support the use of DNA pooling in these species to reduce genotyping costs.

### Cross-species SNP performance with Japanese red seabream and yellowtail kingfish

A small number of Japanese red seabream (*n* = 39) and yellowtail kingfish (*n* = 23) DNA samples were screened over the array. The objective was to evaluate the performance of snapper and silver trevally SNPs on two closely related species within the same family, as it has been performed successfully in other taxa (Montanari *et al*. 2013; Gutierrez *et al*. 2017; Mastrochirico-Filho *et al*. 2021; Peñaloza *et al*. 2021). Almost all red seabream samples were successfully genotyped with snapper SNPs (Supplementary Table 1), even though call rates were visibly lower than in snapper samples (Fig. 6c). This was expected, as the snapper marker probes are more likely to have additional polymorphisms when hybridized with seabream DNA because of the genetic divergence between the two species, causing these samples to fall in between genotypic clusters, as it is typical of OTV SNPs. Evaluation of a random subset of PHR and NMH SNP cluster plots showed that the seabreams often grouped together with snapper samples (Supplementary Figs. 2a and b). Some well-clustered OTV SNPs highlighted the same pattern (Supplementary Fig. 2c). In contrast, others showed a clear separation between red seabream and snapper (Supplementary Fig. 2d), indicating that this array is helpful for examining the genetic diversity between these two species. This was further confirmed by the PCA (Figs. 4a and b). Additionally, while the majority of the SNPs were monomorphic within the red seabream, 3,511 high-quality polymorphic SNPs (PHR, NMH, or OTV) were identified, indicating that this array could be used to also evaluate intra-species genetic diversity to a certain extent. The number of polymorphic SNPs would certainly be of great value for selective breeding programs for red seabream (Murata *et al*. 1996), where parent assignments following mass spawning would be needed. Concerning kingfish, all samples had very low DQC values and genotypes could not be called (Fig. 6a; Supplementary Table 1). This might either be because of a large divergence between trevally and kingfish genomes (which would cause lower DQC values) or because of insufficient DNA quality, and more samples would need to be screened to confirm this.

## Conclusions

Our study clearly demonstrates that multiplexing plant and animal SNPs on the same array and pooling DNA from two distantly related species are possible and efficient. This is a promising solution for cutting costs for high-throughput genotyping in half. As prices for SNP arrays decrease and genotyping platforms start to provide more comprehensive services that include the extraction and handling of DNA from different tissues, this type of array design is becoming more common. The SNP array developed here had a conversion rate that ranged between 56% (mānuka) and 61% (snapper), which enabled a genotyping density suitable for applications in both breeding and conservation strategies. The robust and highly polymorphic SNP markers developed here for each species might be used in the future to design higher efficiency multi-species arrays, as well as being supplemented with new markers.

## Supplementary Material

jkad170_Supplementary_Data

## Data Availability

The SNP marker sequences and their location on the *R. occidentalis*, *Rubus argutus*, *L. scoparium*, *C. auratus*, and *P. georgianus* reference genomes are provided in Supplementary Table 4. The R and python codes used for the evaluation of the genotypic data and the SNP validation are provided in Supplementary File S1. The Python script used to design the snapper circular pedigree plot was deposited in Zenodo: https://doi.org/10.5281/zenodo.7939180. Raw sequencing and genotyping data for *Rubus* spp. are available at the National Center for Biotechnology Information (https://www.ncbi.nlm.nih.gov/) under Bioproject IDs PRJNA1002337 and PRJNA1002481 and the Genome Database for Rosaceae (https://www.rosaceae.org/) under accession number tfGDR1073. Access to raw and analyzed data of mānuka, trevally, and snapper will require permission from the representatives of Māori iwi (tribes) who exercise guardianship for this material according to Aotearoa-NZ’s Treaty of Waitangi and the international Nagoya protocol on the rights of indigenous peoples. Supplemental material available at G3 online.

## Appendix A: Methods for sequencing, variant calling, and SNP filtering, and for SNP validation in *Rubus* spp.

### Sequencing, variant calling, and SNP filtering

*Raspberry*. Data from genotyping experiments produced using reduced-representation GBS were available for seven F_1_*Rubus* subgenus *Idaeobatus* populations, including sequences from the offspring and, where available, the parents and grandparents (Supplementary Table 2). One family was developed at North Carolina State University from a cross between accession NC493 (*Rubus parvifolius* × *R. idaeus* “Cherokee”) and “CW” (*R. idaeus*). GBS data for NC493×CW were retrieved from Jibran *et al*. (2019). The raw VCF file was used, which included 649,597 SNPs for all offspring and parents with no filtering applied. Six families generated at PFR by crossing *R. idaeus* breeding selections consisted of: X14.102 (*n* = 157), X16.015 (*n* = 94), X16.093 (*n* = 47), X16.095 (*n* = 199), X16.109 (*n* = 56), and X16.111 (*n* = 49). For these populations, GBS libraries were prepared and sequenced using the protocol of Jibran *et al*. (2019). Reads were then trimmed of TruSeq Illumina adapters with Trim Galore v0.4.3 (https://github.com/FelixKrueger/TrimGalore), de-multiplexed with *fastq_multx* in ea-utils v1.1.2-806 (https://github.com/ExpressionAnalysis/ea-utils), and aligned to the *R. occidentalis* v3.0 genome (VanBuren *et al*. 2016) with BWA-mem v0.7.17 (Li and Durbin 2009). Variant calling was performed with SAMtools v1.7 (*mpileup*) and bcftools v1.10.2 (multi-allelic-caller) (Danecek *et al*. 2021). For the X14.102, X16.015, and NC493×CW families, INDELs were filtered out, and individuals with >80% missing rate and SNPs with RMS mapping quality <20, DP <10 or >1,000 were removed. The same parameters were used for the other families, except that SNPs were filtered for RMS mapping quality >20, DP >8, and MAF >0.05.

All the above datasets were merged with VCFtools v0.1.14 (Danecek *et al*. 2011) *vcf-merge* to identify and eliminate sites with another SNP within 30 bp up- or down-stream (“thinning”). The list of remaining SNPs was intersected back with each of the four datasets, from which a core of “validated SNPs” was subsequently identified. “Validated SNPs” had no inconsistencies between technical replicates of the same genotype, exhibited <5% Mendelian errors, <80% missing data, and were polymorphic within the families; additionally, individuals with >10% error rate were also removed. Finally, genotypic data for the families X16.093, X16.095, X16.109, X16.111, and NC493×CW were imported into JoinMap v5.0 (VanOoijen 2006) and grouped into LGs with a LOD ≥10; SNPs that were not successfully included into one of the expected seven LGs were eliminated from the “validated SNPs” datasets of those families. As a last filtering step, A/T and C/G SNPs were discarded and a unique list of SNPs from all datasets was obtained. Finally, a total of 859 SNPs, designed on candidate genes controlling sugar content and validated with a KASP assay (Zurn *et al*. 2020a), were added to the merged dataset.

A visual representation of the variant calling and filtering performed in *Rubus* is reported in Supplementary Fig. 3.

*Blackberry*. WGS data for 27 blackberry cultivars and advanced selections from the UArk and USDA-ARS breeding programs (Supplementary Table 8) were used for SNP selection. Samples were sequenced on Illumina HiSeq 2500 to generate from 84.6 to 123.7 million 2 × 150 bp paired-end reads per sample. Raw Illumina reads were processed to remove contaminating sequencing adapters and low-quality reads using the CLC Genomics Workbench v20.0 (Qiagen, Hilden, Germany). Adapter-trimmed, high-quality reads were then mapped to a contig-scale genome assembly of the diploid blackberry “Hillquist” (*R. argutus*) (Worthington *et al*. 2020). Mapping was performed with 90% identity and 90% read coverage parameters using CLC Genomics Workbench. The mapped bam file was sorted using SAMtools v1.11 and the duplicate reads were marked using Sambamba v0.8.0 (Tarasov *et al*. 2015). Variant calling was performed on the deduplicated bam file using Freebayes v1.3.2 (Garrison and Marth 2012). The Freebayes output was further filtered using bcftools v1.11 and custom scripts as follows to obtain high-quality and biologically relevant SNP markers. Only biallelic SNPs (no A/T and C/G) with at least one homozygous individual in the panel, less than 33% missing data, and no other flanking variants in a 30 bp up- or down-stream window were selected. Finally, these SNPs were re-aligned to a new chromosome-length assembly of *R. argutus* (Brůna *et al*. 2022) and only those that mapped to a unique position were selected.

The flanking sequences of the blackberry SNPs were BLAST (v2.6.0) searched against the *R. occidentalis* genome and those of the raspberry SNPs were BLAST searched against the *R. argutus* genome (Brůna *et al*. 2022), with the objective of identifying overlapping SNPs between the two datasets and retaining one. In addition, blackberry SNPs with multiple hits on the raspberry reference genome were discarded, as they could result in erroneous genotypic calls in raspberry individuals.

### SNP validation

Diploid and tetraploid *Rubus* samples from PFR, (USDA-ARS)—NCGR, and UArk were analyzed.

*Diploids*. A total of 477 samples were estimated to be diploid, including 332 from PFR, 143 from NCGR, and two from UArk. These samples were analyzed together in the Axiom Analysis Suite v5.1.1 software. Cluster plots of a random subset of PHR SNPs were observed to verify the quality of the call and make necessary adjustments. Samples with an “allele_deviation_mean” value (i.e. the average of the absolute difference between the log_2_ allele signal estimate and its median across all SNPs) higher than 0.85 were then removed and the remaining samples were re-analyzed. SNPs categorized as PHR and NMH were then verified for consistency in biologically replicated samples (i.e. samples collected twice or more from the same plant). The similarity of duplicated samples was checked by calculating pairwise IBS values in the R package SNPRelate v1.18.1 (Zheng *et al*. 2012). All replicates with an IBS >0.97 were considered truly identical and used to remove markers with inconsistent genotypic calls and identify a subset of robust SNPs to use for follow-up analysis. A PCA was run on the diploid good quality samples using the robust SNPs filtered for MAF >0.05 and LD-pruned with a threshold of 0.2. Additionally, a DAPC was run in the R package adegenet v2.1.2 (Jombart 2008; Jombart *et al*. 2010; Jombart and Ahmed 2011).

*Tetraploids*. The overall number of tetraploid samples analyzed was 739, including 262 from PFR, 66 from NCGR, and 411 from UArk. Summarized signal intensities for all 12,723 *Rubus* SNPs for these samples were obtained in the Axiom Analysis Suite software and then imported into R for dosage calling with the package fitPoly v3.0.0 (Voorrips *et al*. 2011; Zych *et al*. 2019). The command *saveMarkerModels* was used with a *P*-value threshold of 0.9. The dataset was subsequently filtered for missing rate, applying a 20% cut off for both samples and SNPs. Finally, a PCA was run using the R package polymapR v1.1.2 (Bourke *et al*. 2018).

## Appendix B: Methods for sequencing, variant calling, and SNP filtering, and for SNP validation in mānuka

### Sequencing, variant calling, and SNP filtering

The pooled sequencing data from Koot *et al*. (2022) were used for SNP selection. This dataset consisted of 68 pooled populations of mānuka sampled across Aotearoa-NZ. Five gene pools were identified amongst these populations by Koot *et al*. (2022), namely in the NNI, the CSNI, the ECNI, and two gene pools in the SI representing the NESI and SWSI, respectively. The variants called in the 68 pooled populations were filtered using VCFtools v0.1.14 (Danecek *et al*. 2011), by keeping SNPs with MAF >0.05, a mean DP of 100, and no missing data and discarding A/T and C/G SNPs. Additionally, MAFs were calculated and averaged across populations within each gene pool and used to identify SNPs specific to each of the gene pools, as well as to the North Island and SI, respectively. The SNP locations were based on the reference genome of *L. scoparium* “Crimson Glory” (Thrimawithana *et al*. 2019).

### SNP validation

A subset of 264 samples used for pool sequencing and variant detection by Koot *et al*. (2022) was employed for SNP validation. The samples were chosen as representatives of five gene pools: NNI, ECNI, CSNI, NESI, and SWSI (Supplementary Table 6). Population structure was investigated using K-means clustering and a DAPC in the R package adegenet v2.1.2 (Jombart 2008; Jombart *et al*. 2010; Jombart and Ahmed 2011). Weir and Cockerham's pairwise *F_ST_* distances (Weir and Cockerham 1984) and accompanying *P*-values were estimated among gene pools using the R package StAMPP v1.6.3 (Pembleton *et al*. 2013) and applying nboots = 1000, percentage = 95.

## Appendix C: Methods for sequencing, variant calling, and SNP filtering, and for SNP validation in snapper

### Sequencing, variant calling, and SNP filtering

Both parental individuals and six offspring of ten F_1_ families from the PFR snapper breeding program, accounting for a total of 80 samples, were sequenced using the Illumina Novaseq technology. Reads were trimmed using Trimmomatic v0.36 (Bolger *et al*. 2014), then aligned to the *C. auratus* v1.0 male reference genome (Catanach *et al*. 2019) with BWA-mem v0.7.15 (Li and Durbin 2009). Variants were called with three different software [SAMtools v1.7 *mpileup* and bcftools v1.10.2 multi-allelic-caller (Danecek *et al*. 2021); GATK v4.0.3.0 HaplotypeCaller (McKenna *et al*. 2010); and FreeBayes v1.3.1 (Garrison and Marth 2012)] and combined into a final consensus set. SNPs with another variant within 30 bp up- or down-stream were removed (“thinning”), as well as those with >20% missing data, DP >6,493 (= average DP + 3 SDs), and MAF <0.05. Multi-allelic and A/T and C/G SNPs were also discarded. Finally, the remaining SNPs were pruned for LD using bcftools + prune, retaining only four SNPs with *r*^2^ > 0.85 per 100 kb window.

### SNP validation

Genotypes for snapper were called keeping the two batches separated, as a large number of samples were screened (*n* = 2,525 and *n* = 1,719, respectively, for first and second batch). Seabream samples (*n* = 39) were included in the first batch. The two datasets were then merged and only SNPs classified as PHR in both datasets were kept for subsequent analysis. A PCA was run using the R package SNPRelate v1.18.1 (Zheng *et al*. 2012) to examine the genetic diversity between snapper and seabream, as well as the structure of the snapper samples. Additionally, pedigree reconstruction was carried out for a subset of snapper samples corresponding to three separate broodstock lines generated at PFR (broodstock 1: 29 parents, 1,114 offspring; broodstock 2: 35 parents, 965 offspring; broodstock 3: 54 parents, 1,153 offspring). The KING-robust algorithm (Manichaikul *et al*. 2010) in SNPRelate was used to calculate pairwise kinship coefficients (*k*) and IBD0 values between samples. Putative trios were identified as those with the highest ratio of *k*:IBD0 between parent and offspring samples. True trios were then confirmed if they had less than 2% Mendel errors as calculated using the python package scikit-allel v1.3.3 (https://github.com/cggh/scikit-allel).

## Appendix D: Methods for sequencing, variant calling, and SNP filtering, and for SNP validation in trevally

### Sequencing, variant calling, and SNP filtering

The dataset employed here included 17,795,808 SNPs that resulted from the calling and quality filtering performed by Valenza-Troubat *et al*. (2022a) on WGS reads of 13 trevally samples, representing parental individuals of the PFR breeding program, and using the male reference genome for trevally (Catanach *et al*. 2021). As for snapper, this dataset was filtered by removing SNPs that had another polymorphism within 30 bp up- or down-stream (“thinning”), >20% missing data, DP >445 (= average DP + 3 SDs), MAF <0.05, and were multi-allelic or A/T or C/G. The remaining SNPs were then LD-pruned, keeping only five SNPs with *r*^2^ > 0.85 per 100 kb window.

### SNP validation

A subset of 978 trevally samples, representing samples from fish caught in the wild in Aotearoa-NZ and Australia, was analyzed for SNP validation (Supplementary Table 7). SNPs that were classified as PHR and NMH were filtered for MAF >0.05 and LD-pruned with a threshold of 0.2. A PCA was run using the *prcomp* function in R (setting *center* = *TRUE*, *scale.* = *TRUE*) and results were plotted with the factoextra package v1.0.7 (Kassambara and Mundt 2016). Weir and Cockerham's weighted *F_ST_* was estimated for country of origin in SNPRelate v1.18.1 (Zheng *et al*. 2012).
