# Supplementary material for: A multiplexed plant–animal SNP array for selective breeding and species conservation applications
Source: G3 (Bethesda). 2023 Aug 11;13(10):jkad170. doi: 10.1093/g3journal/jkad170 (PMC10542201; doi:10.1093/g3journal/jkad170)
Supplement: jkad170_Supplementary_Data [file jkad170_supplementary_data.zip › File_S1_G3-2023-404167.pdf]

Multi-species SNP array v1.0

Share ••• Star this space

Full scripts

Rubus spp.

- SNP validation in diploid *Rubus* (R)

```
---
title: "Rubus_SNP_chip_validation_diploids_forG3"
output: html_document
---

```{r upload libraries}
library(ggplot2)
library(trio)
library(gdsfmt)
library(SNPRelate)
library(dplyr)
library(graphics)
library(fields)
library(adegenet)
library(colorRamps)
library(plotly)
```

1) Consistency of SNP calls between pairs of known duplicated samples

In Axiom Suite:
- sample QC: DQC>0.82, QCCR>95, allele_deviation_mean<0.85

```{r Filter for PHR and NMH SNPs}
calls <- read.table("AxiomGT1.calls.txt", header = T)

# PHR NMH SNPs only
PHR <- read.table("PolyHighResolution.ps", header = T)
NMH <- read.table("NoMinorHom.ps", header = T)
calls_PHR_NMH <- calls[(calls$probeset_id %in% PHR$probeset_id) | (calls$probeset_id
```

```{r open and check GDS file}
genofile <- snpgdsOpen("Rubus_diploids_PHR_NMH.gds")

read.gdsn(index.gdsn(genofile, "genotype"), start=c(1,1), count=c(5,3))
head(read.gdsn(index.gdsn(genofile, "sample.id")))
head(read.gdsn(index.gdsn(genofile, "snp.id")))
head(read.gdsn(index.gdsn(genofile, "snp.chromosome")))
head(read.gdsn(index.gdsn(genofile, "snp.position")))
```

```{r calcaute IBS values between pairs of samples}

ibs <- snpgdsIBS(genofile, autosome.only = F, remove.monosnp = F)

image.plot(ibs$ibs, col=rev(heat.colors(20)))

ibs_df <- data.frame(ibs$ibs)
colnames(ibs_df) <- ibs$sample.id
```
```

````
rownames(ibs_df) <- ibs$sample.id
head(ibs_df)
```

```{r check IBS values of known duplicated samples}
# BC 64-9-81
BC64.9.81 <- c("EY0052588", "EY0052965")
BC64.9.81_ibs <- ibs_df[rownames(ibs_df) %in% BC64.9.81, colnames(ibs_df) %in% BC64.9
BC64.9.81_ibs
image.plot(as.matrix(BC64.9.81_ibs), col=rev(heat.colors(20)), axes=F, zlim=range(unl
axis(1, at=seq(0,1), labels=BC64.9.81)
axis(2, at=seq(0,1), labels=BC64.9.81)
title(main = "BC 64-9-81")

# Glen Ample
Glen_Ample <- c("EY0052601", "EY0052978")
Glen_Ample_ibs <- ibs_df[rownames(ibs_df) %in% Glen_Ample, colnames(ibs_df) %in% Glen
Glen_Ample_ibs
image.plot(as.matrix(Glen_Ample_ibs), col=rev(heat.colors(20)), axes=F, zlim=range(un
axis(1, at=seq(0,1), labels=Glen_Ample)
axis(2, at=seq(0,1), labels=Glen_Ample)
title(main = "Glen Ample")

# R. spectabilis Gibbs Lake
R.spectabilis_Gibbs_Lake <- c("EY0052345", "EY0052353", "EY0052369")
R.spectabilis_Gibbs_Lake_ibs <- ibs_df[rownames(ibs_df) %in% R.spectabilis_Gibbs_Lake
R.spectabilis_Gibbs_Lake_ibs
image.plot(as.matrix(R.spectabilis_Gibbs_Lake_ibs), col=rev(heat.colors(20)), axes=F,
axis(1, at=seq(0,1, by=0.5), labels=R.spectabilis_Gibbs_Lake)
axis(2, at=seq(0,1, by=0.5), labels=R.spectabilis_Gibbs_Lake)
title(main = "R. spectabilis Gibbs Lake")

# Wakefield
Wakefield <- c("EY0052562", "EY0052723", "EY0052863", "EY0052952", "WG6796605.DNA_B07
Wakefield_ibs <- ibs_df[rownames(ibs_df) %in% Wakefield, colnames(ibs_df) %in% Wakefi
Wakefield_ibs
image.plot(as.matrix(Wakefield_ibs), col=rev(heat.colors(20)), axes=F, zlim=range(unl
axis(1, at=seq(0,1, by=0.25), labels=Wakefield)
axis(2, at=seq(0,1, by=0.25), labels=Wakefield)
title(main = "Wakefield")
```

Only known duplicated samples with IBS > 0.97 will be used.
Wakefield: there are two groups of duplicated: EY0052562 = EY0052723 = WG6796605.DNA_

```{r find and remove SNPs with inconsistent genotypes between dup samples}
known_dup <-  c("EY0052588", "EY0052965", "EY0052601", "EY0052978", "EY0052562", "EY0

dup<-data.frame(which(ibs_df > 0.97, arr.ind = T), row.names = NULL)
dup_row <- ibs$sample.id[dup$row]
dup_col <- ibs$sample.id[dup$col]
dup<- data.frame(cbind(dup_row, dup_col)) %>%
  filter(dup_row %in% known_dup & dup_col %in% known_dup) %>%
  filter(dup_row != dup_col)

SNP_dup_errors <- data.frame(calls_PHR_NMH[, 1])
colnames(SNP_dup_errors) <- "probeset_id"
for (i in rownames(dup)) {
````

```
  sample.1 <- dup[i, 1]
  sample.2 <- dup[i, 2]
  calls_dup <- calls_PHR_NMH[, (colnames(calls_PHR_NMH) == "probeset_id" | colnames(c
  calls_dup$x1 <- case_when(calls_dup[,2] == calls_dup[,3] ~ "ok",
                            calls_dup[,2] == "-1" ~ "ok",
                            calls_dup[,3] == "-1" ~ "ok")
  calls_dup$x1[is.na(calls_dup$x1)] <- "wrong"
  colnames(calls_dup)[4] <- i
  SNP_dup_errors <- left_join(SNP_dup_errors, calls_dup, by="probeset_id")
}
head(SNP_dup_errors)

SNP_dup_errors$count <- apply(SNP_dup_errors, 1, function(x) length(which(x == "wrong
table(SNP_dup_errors$count)

good_SNPs_1 <- SNP_dup_errors[SNP_dup_errors$count == 0,]$probeset_id
```
6,885 SNPs with 0 error (i.e. no inconsistencies within each pair of duplicates) are

```{r close GDS}
snpgdsClose(genofile)
```


1) Structure analysis

```{r open and check GDS file good SNPs}
genofile <- snpgdsOpen("Rubus_diploids_good_SNPs.gds")

read.gdsn(index.gdsn(genofile, "genotype"), start=c(1,1), count=c(5,3))
head(read.gdsn(index.gdsn(genofile, "sample.id")))
head(read.gdsn(index.gdsn(genofile, "snp.id")))
head(read.gdsn(index.gdsn(genofile, "snp.chromosome")))
head(read.gdsn(index.gdsn(genofile, "snp.position")))
```

```{r MAF}
maf <- snpgdsSNPRateFreq(genofile, with.id = T)
SNP_maf <- cbind(data.frame(maf$snp.id), data.frame(maf$MinorFreq))

ggplot(SNP_maf, aes(maf.MinorFreq)) +
  geom_histogram()
```

```{r MAF filtering and LD pruning}
snpset <- snpgdsLDpruning(genofile, ld.threshold=0.2,
                          snp.id = good_SNPs_1_df$probeset_id, autosome.only = F,
                          maf = 0.05,
                          remove.monosnp=F, method = "corr")

snpset.id <- unlist(unname(snpset))
length(snpset.id)
```

```{r PCA}
pca <- snpgdsPCA(genofile, snp.id=snpset.id, autosome.only = F)

# variance proportion (%)
pc.percent <- pca$varprop*100
```

````
head(round(pc.percent, 2))
```

```{r import sample attributes}
sample_attributes <- read.csv("Rubus_2x_Samples_passed.csv", header = T)
colnames(sample_attributes)[1] <- "sample.id"
samples_pca <- data.frame(pca$sample.id)
colnames(samples_pca) <- "sample.id"
pop <- left_join(samples_pca, sample_attributes, by="sample.id")

```

```{r PCA plot colored by repository}

# Color by repository

#data.frame from uploaded PC table
tab <- data.frame(sample.id = PCs$V1,
                  pop = factor(pop$Plant.batch.ID),
                  EV1 = PCs$V2,    # the first eigenvector
                  EV2 = PCs$V3,    # the second eigenvector
                  stringsAsFactors = FALSE)

tab$pop <- gsub("PFR raspberry germplasm", "PFR", tab$pop)

ggplot(tab, aes(x=EV1, y=EV2, color=pop)) +
  geom_point(alpha=0.8,size=3,) +
  theme_classic() +
  theme(legend.title = element_blank(), legend.text = element_text(size=12),
        axis.title = element_text(size = 12)) +
  xlab("PC1 25.44%") + ylab("PC2 7.24%") +
  scale_color_manual(values = c("black", "red"))
```

```{r PCA plot colored by species}
# Color by species

pop$Plant.species[is.na(pop$Plant.species)] <- "unknown"

#data.frame from uploaded PC table
tab <- data.frame(sample.id = PCs$V1,
                  pop = factor(pop$Plant.species),
                  EV1 = PCs$V2,    # the first eigenvector
                  EV2 = PCs$V3,    # the second eigenvector
                  stringsAsFactors = FALSE)


tab %>% ggplot() +
  geom_point(data = . %>% filter(pop != "unknown"), aes(x=EV1, y=EV2, color=pop), alp
  geom_point(data = . %>% filter(pop == "unknown"), aes(x=EV1, y=EV2), color="grey",
  theme_classic() +
  theme(legend.title = element_blank(), legend.text = element_text(size=12),
        axis.title = element_text(size = 12)) +
  xlab("PC1 25.44%") + ylab("PC2 7.24%") +
  scale_color_manual(values = c("R. crataegifolius" = "#5eadff",
                                "R. idaeus" = "#3c0068",
                                "R. lasiostylus" = "#a1006a",
                                "R. neglectus" = "#9d3700",
````

````
                                "R. occidentalis" = "#ffae66",
                                "R. spectabilis" = "#4f5300",
                                "R. strigosus" = "#d3db7d",
                                "R. trivialis" = "#2e8200",
                                "Rubus hybrid" = "#01a95c",
                                "unknown" = "grey"))
```

```{r convert GDS to BED}
snpgdsGDS2BED(genofile, bed.fn="Rubus_diploids_robust_SNPs_LD0.8", snp.id=snpset.id)
```

```{r close GDS}
snpgdsClose(genofile)
```

```{bash create raw file to import in adegenet}
module load plink
cd /workspace/hrpsym/Rubus/SNPChip_Genotyping1/
plink --bfile Rubus_diploids_robust_SNPs_LD0.8 --make-bed --out Rubus_diploids_robust
plink --bfile Rubus_diploids_robust_SNPs_LD0.8_new --allow-extra-chr --recode A --out
```

```{r import SNP data from plink raw file to genlight object}
snp_data<-read.PLINK("Rubus_diploids_robust_SNPs_LD0.8_new.raw")
snp_data
```

```{r fidentify number of clusters}
grp <- find.clusters(snp_data, n.pca = 200, max.n.clust=50)

# Choose the number of clusters (>=2: 50
```

```{r display BIC values}
grp$Kstat
```
The elbow of the BIC curve is at 13, the lowest BIC is at 11

```{r Repeat find.cluster choosing 11 clusters}
grp_11 <- find.clusters(snp_data, n.pca = 200, n.clust = 11)
```

```{r DAPC to chose number of PCs and DAs}
dapc1 <- dapc(snp_data, grp_11$grp)
```
PCs to retain: 100
discriminant functions to retain 4

```{r DAPC}
dapc2 <- dapc(snp_data, grp_11$grp, n.pca = 100, n.da = 4)
dapc2
```
- the slots ind.coord and grp.coord contain the coordinates of the individuals and of
- Contributions of the alleles to each discriminant function are stored in the slot v
- Eigenvalues are stored in eig

```{r 3D plot DAPC colored by cluster asssignement}
````

```
dapc2_coord<-data.frame(dapc2$ind.coord)
dapc2_coord_assign<-data.frame(cbind(dapc2_coord, dapc2$assign))

plot_ly(x=dapc2_coord_assign$LD1, y=dapc2_coord_assign$LD2, z=dapc2_coord_assign$LD3,
        type="scatter3d", mode="markers",
        color = dapc2_coord_assign$dapc2.assign, colors = primary.colors(11)) %>%
  layout(scene = list(xaxis=list(title = 'LD1'), yaxis=list(title = 'LD2'), zaxis=lis
```

```{r 3D plot_DAPC colored by repository}
dapc2_coord_assign$sample.id <- rownames(dapc2_coord_assign)
dapc2_coord_assign_germplasm <- left_join(dapc2_coord_assign, pop, by="sample.id")

dapc2_coord_assign_germplasm$colors <- case_when(dapc2_coord_assign_germplasm$Plant.b
                                                 dapc2_coord_assign_germplasm$Plant.b
                                                        )

plot_ly(x=dapc2_coord_assign_germplasm$LD1, y=dapc2_coord_assign_germplasm$LD2, z=dap
        type="scatter3d", mode="markers",
        color = as.factor(dapc2_coord_assign_germplasm$colors), colors = c("black", '
  layout(scene = list(xaxis=list(title = 'LD1'), yaxis=list(title = 'LD2'), zaxis=lis
```
```

- fitpoly for SNP dosage calls in tetraploid *Rubus* (R)

````
---
title: "fitpoly"
output: html_document

```{r setup, include=FALSE}
knitr::opts_chunk$set(echo = TRUE)
```

```{r start}
library(fitPoly)
library(fitPolyTools)
```

```{r Read and convert the array data}
datAX <- readAxiomSummary(AXdata="AxiomGT1.summary.txt", out=NA)
head(datAX)
length(unique(datAX$MarkerName))
# 12,723
length(unique(datAX$SampleName))
# 739
```

```{r run models}
saveMarkerModels(ploidy=4,
                 data=datAX,
                 p.threshold=0.9,
                 filePrefix="A",
                 rdaFiles=TRUE,
                 plot="fitted",
                 ncores=1)
```
````
````

```

```{r load and look at results}
load("A_scores.RData")
head(scores)

load("A_models.RData")
head(modeldata)
```

```{r filter for SNPs with successfully fitted models}
modeldata$message <- as.character(modeldata$message)
View(unique(modeldata$message))

passed_SNPs <- as.vector(modeldata[which(!(startsWith(modeldata$message, "rejected"))
                                            !(endsWith(modeldata$message, "no converger

scores_passed <- scores[which(scores$MarkerName %in% passed_SNPs),]
head(scores_passed)
length(unique(scores_passed$MarkerName))
#4872
```

```{r draw XY plots}
XYgeno <- combineFiles(XYdata=datAX, scores=scores)
drawXYplots(dat=XYgeno, markers = passed_SNPs,
            out="XY_plots/XYgeno",
            genocol=get.genocol(ploidy=4),
            ploidy=4)
```
```

- SNP validation in tetraploid *Rubus* (R)

```
---
title: "Rubus_4x_check_forG3"
output: html_document
---

```{r upload libraries}
library(polymapR)
library(ggplot2)
library(dplyr)
library(tidyr)
```

```{r data import}
ALL_dosages <- read.table("A_scores_wide.txt",
                          stringsAsFactors = FALSE,
                          row.names = 1,
                          header = T)
head(ALL_dosages)
class(ALL_dosages)
# data.frame
dim(ALL_dosages)

sample_names <- colnames(ALL_dosages)
sample_names_fixed <- substr(sample_names, 6, 22)
numbers <- seq(1,length(sample_names_fixed), by=1)
```
```

```
sample_names_numbers <- data.frame(rbind(numbers, sample_names_fixed))

colnames(ALL_dosages) <- numbers
head(ALL_dosages)
```

```{r create matrix}
ALL_dosages <- as.matrix(ALL_dosages)
class(ALL_dosages)
# matrix
```

```{r remove individuals wiht missing data}
NAcounts_id <- apply(ALL_dosages, 2, function(x) sum(is.na(x)))

NAtab_id <- data.frame(cbind(colnames(ALL_dosages), NAcounts_id), row.names = NULL)
NAtab_id$NAcounts_id <- as.numeric(NAtab_id$NAcounts_id)

# 20% missing rate = 4872*0.2 = 974.4

ALL_dosages2 <- ALL_dosages[, colnames(ALL_dosages) %in% NAtab_id[NAtab_id$NAcounts_i
dim(ALL_dosages2)
```

```{r remove markers wiht missing data}
NAcounts_m <- apply(ALL_dosages2, 1, function(x) sum(is.na(x)))

NAtab_m <- data.frame(cbind(rownames(ALL_dosages2), NAcounts_m), row.names = NULL)
NAtab_m$NAcounts_m <- as.numeric(NAtab_m$NAcounts_m)

# 20% missing rate = 666*0.2 = 133.2


ALL_dosages3 <- ALL_dosages2[rownames(ALL_dosages2) %in% NAtab_m[NAtab_m$NAcounts_m <
dim(ALL_dosages3)
```
4,388 marekres remained
666 individuals remained

```{r import sample attributes}
sample_attr_all <- read.csv("All_samples.csv")

sample_names_numbers_t <- data.frame(t(sample_names_numbers))
sample_names_numbers_t3 <- sample_names_numbers_t[sample_names_numbers_t$numbers %in%
colnames(sample_names_numbers_t3)[2] <- "Sample.Filename"

sample_attr <- left_join(sample_names_numbers_t3, sample_attr_all, by="Sample.Filenam

unique(sample_attr$Plant.batch.ID)
```

```{r PCA}
PCAdata <- PCA_progeny2(dosage_matrix = ALL_dosages3)


Repository_colors <- sample_attr$Plant.batch.ID
Repository_colors[Repository_colors == "Uark"] <- "yellow2"
Repository_colors[Repository_colors == "NCGR"] <- "violet"
```

```
Repository_colors[Repository_colors == "PFR raspberry germplasm"] <- "turquoise2"

plot(PCAdata$scores[,1], PCAdata$scores[,2],
     xlab="PC1", ylab="PC2",
     col=Repository_colors, pch=16)
legend("topleft", legend=c("UArk", "NCGR", "PFR"),
       col=c("yellow2", "violet", "turquoise2"), lty=1, cex=1)


data.frame(cbind(PCAdata$scores, sample_attr$Plant.batch.ID)) %>%
  mutate_at(c("PC1", "PC2"), as.numeric) %>%
  rename(Repository = V3) %>%
  ggplot(aes(x=PC1, y=PC2, color=Repository)) +
  geom_point(alpha=0.8,size=3) +
  theme_classic() +
  theme(legend.title = element_blank(), legend.text = element_text(size=12),
        axis.title = element_text(size = 12)) +
  xlab("PC1 29.90%") + ylab("PC2 7.65%") +
  scale_color_manual(values = c("UArk" = "yellow2", "NCGR" = "violet", "PFR raspberry
```
```

### Mānuka

- SNP validation in mānuka (R)

```
---
title: "Manuka_SNPchip_analysis"
output: pdf_document

```{r start}
library(data.table)
library(adegenet)
library(ggplot2)
library(StAMPP)
library(gplots)
```

```{r input data}
samples<-fread("Manuka_SNParray.csv")
```

```{r DAPC}
grp<-find.clusters(samples,max.n=20, n.pca=250, scale=FALSE,
choose.n.clust=FALSE,criterion="min" )
dapc<-dapc(samples, grp = grp$grp, n.pca = 100, n.da = 100,scale = FALSE, var.contrib
dapc_optim<-optim.a.score(dapc)
dapc<-dapc(samples, grp = grp$grp,n.da=100,n.pca=dapc_optim$best)
dapc_coordinates<-as.data.frame(dapc$ind.coord)
p <- plot_ly(dapc_coordinates, type = "scatter3d",x = ~LD1 , y = ~LD2, z= ~LD3, mode =
p
```

```{r Fst}
df <- stamppConvert(samples, type = "r")
fst <- stamppFst(df, nboots = 1000, percent = 95, nclusters = 1)
fst_m<-Matrix::forceSymmetric(fst$Fsts,uplo="L")
heatmap.2(fst_m,dendrogram = "row",distfun = dist,
```

```
hclustfun = hclust,
reorderfun = function(d, w) reorder(d, w),keysize=1, trace="none", cexRow=1.2, cexCol=
```
```

### Snapper

- PCA for snapper and seabream (R)

```
---
title: "Seabream_PCA_forG3"
output: html_document

```{r upload libraries}
library(ggplot2)
library(gdsfmt)
library(SNPRelate)
library(dplyr)
library(graphics)
library(fields)
library(adegenet)
library(colorRamps)
library(RColorBrewer)
library(cowplot)
library(plotly)
```

In Axiom Suite:
- 1st batch and 2nd batch analysed separately
- sample QC: DQC>0.82, QCCR>95

```{r combine SNP categories from 1st and 2nd batch}
snapper_1st_batch <- read.table("SNP_categories_1stbatch.txt", header = T)
snapper_2nd_batch <- read.table("SNP_categories_2ndbatch.txt", header = T)

snapper_all <- inner_join(snapper_1st_batch, snapper_2nd_batch, by="probeset_id")
colnames(snapper_all)[2] <- "ConversionType_1st_batch"
colnames(snapper_all)[3] <- "ConversionType_2nd_batch"

table(snapper_all$ConversionType_1st_batch)
table(snapper_all$ConversionType_2nd_batch)
```

```{r combine gneotypic calls from two batches and filter for PHR NMH}
calls1 <- read.table("AxiomGT1.calls_1stbatch.txt", header = T)
calls2 <- read.table("AxiomGT1.calls_2ndbatch.txt", header = T)

calls <- inner_join(calls1, calls2, by="probeset_id")

# PHR NMH SNPs only
calls_PHR_NMH <- calls[(calls$probeset_id %in% snapper_all[(snapper_all$agreement ==
```

```{r open and check GDS file}
genofile <- snpgdsOpen("Snapper_Seabream_1st&2nd_batch.gds")

read.gdsn(index.gdsn(genofile, "genotype"), start=c(1,1), count=c(5,3))
head(read.gdsn(index.gdsn(genofile, "sample.id")))
```

````
head(read.gdsn(index.gdsn(genofile, "snp.id")))
head(read.gdsn(index.gdsn(genofile, "snp.chromosome")))
head(read.gdsn(index.gdsn(genofile, "snp.position")))
```

## Structure analysis

```{r MAF}
maf <- snpgdsSNPRateFreq(genofile, with.id = T)
SNP_maf <- cbind(data.frame(maf$snp.id), data.frame(maf$MinorFreq))

ggplot(SNP_maf, aes(maf.MinorFreq)) +
  geom_histogram()
```

```{r MAF filtering and LD pruning}
snpset <- snpgdsLDpruning(genofile, ld.threshold=0.2,
                          autosome.only = F,
                          maf = 0.05,
                          remove.monosnp=F, method = "corr")

snpset.id <- unlist(unname(snpset))
length(snpset.id)
```

```{r PCA}
pca <- snpgdsPCA(genofile, snp.id=snpset.id, autosome.only = F)

# variance proportion (%)
pc.percent <- pca$varprop*100
head(round(pc.percent, 2))
```

```{r import sample attributes}
sample_attributes <- read.csv("Samples_atteributes.csv", header = T)
colnames(sample_attributes)[1] <- "sample.id"
samples_pca <- data.frame(pca$sample.id)
colnames(samples_pca) <- "sample.id"
pop <- left_join(samples_pca, sample_attributes, by="sample.id")

unique(pop$Fish.Population.ID)
```

```{r PCA plots colored by fish population}
# make a data.frame
tab <- data.frame(sample.id = pca$sample.id,
                  pop = factor(pop$Fish.Population.ID),
                  EV1 = pca$eigenvect[,1],
                  EV2 = pca$eigenvect[,2],
                  EV3 = pca$eigenvect[,3],
                  EV4 = pca$eigenvect[,4],
                  stringsAsFactors = FALSE)

ggplot(tab %>%
         arrange(desc(pop)),
       aes(x = EV1, y = EV2, color=pop)) +
  geom_point(alpha=0.8) +
  scale_color_manual(values = c("black", "hotpink3", "lightgoldenrod3", "mediumseagre
````

```
  #scale_color_brewer(palette = "Set2", direction = -1) +
  guides(color=guide_legend(title="Population")) +
  xlab("PC1 7.86%") + ylab("PC2 6.59%") +
  theme_classic() +
  theme(legend.title = element_blank(), legend.text = element_text(size=12),
        axis.title = element_text(size = 12))

ggplot(tab %>%
         arrange(desc(pop)),
       aes(x = EV3, y = EV4, color = pop)) +
  geom_point(alpha=0.8) +
  scale_color_manual(values = c("black", "hotpink3", "lightgoldenrod3", "mediumseagre
  #scale_color_brewer(palette = "Set2", direction = -1) +
  guides(color=guide_legend(title="Population")) +
  xlab("PC3 3.81%") + ylab("PC4 2.73%") +
  theme_classic() +
  theme(legend.title = element_blank(), legend.text = element_text(size=12),
        axis.title = element_text(size = 12))
```

```{r close GDS}
snpgdsClose(genofile)
```
```

- Pedigree reconstruction for snapper (R)

```
if (!requireNamespace("BiocManager", quietly = TRUE))
  install.packages("BiocManager")
BiocManager::install("SNPRelate")

library(ggplot2)
library(gdsfmt)
library(SNPRelate)
library(dplyr)
library(graphics)
library(fields)
library(adegenet)
library(colorRamps)
library(plotly)

call_SNPRelate <- function(
  snp_marker_info_path,
  raw_genotype_file,
  GDS_output_file, # Created in pipeline
  ibd_kinship_output_path, # Created in pipeline
  ibs0_output_path # Created in pipeline
) {

  array_SNPinfo<-read.table(snp_marker_info_path, header = T)
  calls<-read.table(raw_genotype_file, header = T)

  rownames(calls) <- calls$sampleID
  calls <- select(calls, -sampleID)
  calls<-data.matrix(calls)

  snpgdsCreateGeno(GDS_output_file,
```
```

```
                  genmat = calls,
                  sample.id = rownames(calls),
                  snp.id = array_SNPinfo$probeset_id,
                  snp.chromosome = array_SNPinfo$chr,
                  snp.position = array_SNPinfo$position,
                  snpfirstdim = F)

  genofile <- snpgdsOpen(GDS_output_file)
  snpgdsSummary(genofile, show = T)

  ibd.robust <- snpgdsIBDKING(genofile, autosome.only = F,type=c("KING-robust", "KING-

  # Writing kinship df
  ibd_kinship_df <- data.frame(ibd.robust[["kinship"]])
  colnames(ibd_kinship_df) <- ibd.robust[["sample.id"]]
  rownames(ibd_kinship_df) <- ibd.robust[["sample.id"]]

  write.csv(ibd_kinship_df, file=ibd_kinship_output_path)

  # Writing IBS0 df
  ibs0_df <- data.frame(ibd.robust[["IBS0"]])
  colnames(ibs0_df) <- ibd.robust[["sample.id"]]
  rownames(ibs0_df) <- ibd.robust[["sample.id"]]

  write.csv(ibs0_df, file=ibs0_output_path)

}
####### Calling the function


call_SNPRelate(
  "markerinfo_file.txt",
  "genotype_file.raw",
  "snprelate_input.gds", # Created in pipeline
  "IBD_kinship_output.txt", # Created in pipeline
  "IBS0_output.txt" # Created in pipeline
)


```

- Mendel test in snapper (Python)

```
import toml
import pandas
import pathlib
import numpy
import allel

from dataclasses import dataclass
from typing import List


@dataclass
class Sample:
    population_identifier: str = None
    genotype_array_identifier: str = None
    parent_population: str = None
```

```python
    genotype_file: str = None
    species_identifier: str = None
    batch_id: str = None
    labogena_batch: str = None
    fish_identifier: str = None
    previous_sampleID: str = None
    generation: str = None
    possible_parent_genotype_ids: List[str] = None
    genotype_array: numpy.ndarray = None
    marker_ids: numpy.ndarray = None

    def __repr__(self) -> str:
        return f"{self.__class__.__name__}(Population: {self.population_identifier},

    def return_parent_samples(self, parent_sample_list: List[None]) -> list:
        """
        Takes a list of Sample objects and extract the genotype identifiers of fish c
        parent population
        """

        parent_samples = [
            parent
            for parent in parent_sample_list
            if parent.genotype_array_identifier in self.possible_parent_genotype_ids
        ]

        return parent_samples



@dataclass
class Trio:
    offspring_sample: Sample = None
    parents: List[Sample] = None
    mendel_error_threshold: float = 0.02

    @property
    def mendelian_error(self) -> float:
        """ """
        parent1_genotype = self.parents[0].genotype_array
        parent2_genotype = self.parents[1].genotype_array
        parent_genotypes = parent1_genotype.concatenate([parent2_genotype], axis=1)
        mendel_error_array = allel.mendel_errors(
            parent_genotypes, self.offspring_sample.genotype_array
        )

        snps_with_errors = [x for x in mendel_error_array if x != 0]

        error = len(snps_with_errors) / len(mendel_error_array)

        return error

    @property
    def passes_threshold(self) -> bool:
        """ """

        if self.mendelian_error < self.mendel_error_threshold:
            return True
```

```
        else:
            return False


root = pathlib.Path.cwd().resolve()
input_dir = root / "input_data"
output_dir = root / "output_data"


META_DATA_PATH = input_dir / "Neslon_SNA_meta_library.toml"
GENOTYPES_PATH = input_dir / "SNA_Genotypes.npz"
KINSHIP_PATH = input_dir / "SNA_IBD_kinship_output.txt"
IBS0_PATH = input_dir / "SNA_IBS0_output.txt"



META_DATA = toml.load(META_DATA_PATH)
_GENOTYPES = numpy.load(GENOTYPES_PATH)
ORDERED_MARKERS = _GENOTYPES["ordered_markers"]
GENOTYPES = {}

offspring_populations = [
        "Class15_C11_parents",
        "Class15_WT_parents",
        "Class15_C10_parents",
    ]
parent_populations = ["Class10", "Class11", "Nelson_F0_snapper"]


# Loading SNPRelate Output files
kinship_df = pandas.read_csv(KINSHIP_PATH, sep=",", index_col=0)
ibs0_df = pandas.read_csv(IBS0_PATH, sep=",", index_col=0)
ratio_df = kinship_df / ibs0_df

for entry in _GENOTYPES:
    if entry != "ordered_markers":
        GENOTYPES[entry] = allel.GenotypeArray(_GENOTYPES[entry], dtype="i1")


if __name__ == "__main__":
    MIN_KINSHIP = 0.1
    MAX_IBS0 = 0.02
    N_MATCHES = 2
    MENDEL_ERROR_THRESHOLD = 0.02

    trios = []

    # List of sample identifiers in these dataframes
    genotype_ids_present = ratio_df.columns.values

    parent_samples = [
        Sample(genotype_array=GENOTYPES[key], marker_ids=ORDERED_MARKERS,**sample_met
        for key, sample_meta_dictionary in META_DATA.items()
        if key in GENOTYPES
        and sample_meta_dictionary["population_identifier"] in parent_populations
        and sample_meta_dictionary["genotype_array_identifier"] in genotype_ids_prese
    ]

```

```
    offspring_samples = [
        Sample(genotype_array=GENOTYPES[key], marker_ids=ORDERED_MARKERS,**sample_met
        for key, sample_meta_dictionary in META_DATA.items()
        if key in GENOTYPES
        and sample_meta_dictionary["population_identifier"] in offspring_populations
        and sample_meta_dictionary["genotype_array_identifier"] in genotype_ids_prese
    ]

    """
    Finding parent matches with offspring that have the highest ratio of kinship:IBS0
    and which have kinship and IBS0 Values that meet required conditions
    """
    offspring_ids = [sample.genotype_array_identifier for sample in offspring_samples
    parent_ids = [sample.genotype_array_identifier for sample in parent_samples]

    # Restricting to parent - offspring comparisons (excluding parent-parent comparis
    kinship_df = kinship_df.loc[parent_ids, offspring_ids]
    ibs0_df = ibs0_df.loc[parent_ids, offspring_ids]
    ratio_df = ratio_df.loc[parent_ids, offspring_ids]

    for offspring in offspring_samples:
        best_matches = ratio_df[offspring.genotype_array_identifier].nlargest(N_MATCH
        best_matches = list(best_matches.index)
        offspring.possible_parent_genotype_ids = []

        for match in best_matches:
            condition1 = ibs0_df.loc[match][offspring.genotype_array_identifier] < MA
            condition2 = kinship_df.loc[match][offspring.genotype_array_identifier] >


            if condition1 and condition2:
                offspring.possible_parent_genotype_ids.append(match)

    """
    For all offspring that have potential parent matches -> calculate mendel errors
    and check if this error rate is below threshold error rates.
    """
    for offspring in offspring_samples:
        if len(offspring.possible_parent_genotype_ids) == 2:
            trio = Trio(
                offspring_sample=offspring,
                parents=offspring.return_parent_samples(parent_samples),
                mendel_error_threshold=MENDEL_ERROR_THRESHOLD,
            )
            trios.append(trio)

    print(f"Number of Trios detected: {len(trios)}")
    print(f"Number of trios that pass threshold: {len([trio for trio in trios if tric

```

Trevally

- SNP validation in trevally (R)

```
---
title: "Trevally_SNP_chip_analysis_forG3"
output: html_document
---
```

```
```{r set-up}
library(ggplot2)
library(gdsfmt)
library(SNPRelate)
library(dplyr)
library(graphics)
library(fields)
library(colorRamps)
library(RColorBrewer)
library(cowplot)
library(plotly)
library(factoextra)
```

In Axiom Suite:
- sample QC: DQC>0.82, QCCR>95

```{r filter for PHR and NMH SNPs}
calls <- read.table("AxiomGT1.calls.txt", header = T)

# PHR NMH SNPs only
PHR <- read.table("PolyHighResolution.ps", header = T)
NMH <- read.table("NoMinorHom.ps", header = T)

calls_PHR_NMH <- calls[(calls$probeset_id %in% PHR$probeset_id | calls$probeset_id %i
```

```{r open and check GDS file}
genofile <- snpgdsOpen("Trevally_samples.gds", readonly = F)

read.gdsn(index.gdsn(genofile, "genotype"), start=c(1,1), count=c(5,3))
head(read.gdsn(index.gdsn(genofile, "sample.id")))
head(read.gdsn(index.gdsn(genofile, "snp.id")))
head(read.gdsn(index.gdsn(genofile, "snp.chromosome")))
head(read.gdsn(index.gdsn(genofile, "snp.position")))
```

Structure analysis

```{r MAF}
maf <- snpgdsSNPRateFreq(genofile, with.id = T)
SNP_maf <- cbind(data.frame(maf$snp.id), data.frame(maf$MinorFreq))

ggplot(SNP_maf, aes(maf.MinorFreq)) +
  geom_histogram()
```

```{r filter for MAF and LD pruning}
snpset <- snpgdsLDpruning(genofile, ld.threshold=0.2,
                          autosome.only = F,
                          maf = 0.05,
                          remove.monosnp=F, method = "corr")

snpset.id <- unlist(unname(snpset))
length(snpset.id)
```
```

```
```{r add country info}
sample_attributes <- read.csv("Sample_details.csv", header = T)

sample_list <- data.frame(read.gdsn(index.gdsn(genofile, "sample.id")))
colnames(sample_list) <- "sampl.id"
sample_list_pop <- left_join(sample_list, sample_attributes[, c("sampl.id", "country'

samp.annot <- data.frame(sample_list_pop$country)
add.gdsn(genofile, "sample.annot", samp.annot)
read.gdsn(index.gdsn(genofile, "sample.annot"))
```

```{r Fst}
country <- as.factor(read.gdsn(index.gdsn(genofile, "sample.annot/sample_list_pop.cou
v <- snpgdsFst(genofile, population=country, method="W&C84", autosome.only = F)

v$Fst        # Weir and Cockerham weighted Fst estimate
v$MeanFst    # Weir and Cockerham mean Fst estimate
```

```{r close GDS}
snpgdsClose(genofile)
```

```{r PCA}
genotype_dat <- data.frame(t(calls_PHR_NMH[calls_PHR_NMH$probeset_id %in% snpset.id,-

PCA = prcomp(genotype_dat[ , which(apply(genotype_dat, 2, var) != 0)],
             retx = TRUE,
             center = TRUE,
             scale. = TRUE
             )
```

```{r basic PCA plot}
plot(PCA)
screeplot(PCA, type = "line", main = "Scree plot")

# variance proportion (%)
pc.percent <- (PCA$sdev^2/sum(PCA$sdev^2))*100
head(round(pc.percent, 2))
```

```{r PCA plot colored by country}
fviz_pca_biplot(PCA, col.ind = sample_attributes$country, geom.ind = "point",
                label = "none", invisible = "var",
                palette = "jco",
                addEllipses = TRUE, axes.linetype = "blank",
                legend.title = "Country",
                xlab = "PC1 3.30%", ylab = "PC2 0.39%") +
  theme(panel.grid = element_blank(),
        axis.line = element_line(colour = "black"),
        plot.title = element_blank())
```
```

All species

- Sample pooling evaluation (R)

```
---
title: "Sample_pooling_forG3"
output: html_document
---

```{r start}
library(ggplot2)
library(dplyr)
library(gridExtra)
```

```{r  input data}
table <- read.csv("SNP_array_Summary_sample_pooling.csv")
```

```{r DQC boxplot}
table %>% ggplot() +
  geom_boxplot(data = . %>% filter(Plant.species == "Rubus"),
               aes(x=Plant.species, y=Rubus.DQC, color=Plant.species)) +
  geom_boxplot(data = . %>% filter(Plant.species == "Mānuka"),
               aes(x=Plant.species, y=Mānuka.DQC, color=Plant.species)) +
  geom_boxplot(data = . %>% filter(Fish.species == "Snapper"),
               aes(x=Fish.species, y=Snapper.DQC, color=Fish.species)) +
  geom_boxplot(data = . %>% filter(Fish.species == "Trevally"),
               aes(x=Fish.species, y=Trevally.DQC, color=Fish.species)) +
  geom_boxplot(data = . %>% filter(Fish.species == "Seabream"),
               aes(x=Fish.species, y=Snapper.DQC, color=Fish.species)) +
  geom_boxplot(data = . %>% filter(Fish.species == "Kingfish"),
               aes(x=Fish.species, y=Trevally.DQC, color=Fish.species)) +
  xlab("Species") + ylab("DQC") +
  theme(legend.position = "none", axis.title = element_text(size = 20), axis.text = e
  geom_hline(yintercept=0.82, size=0.2)
```

```{r QCCR boxplot}
table %>% ggplot() +
  geom_boxplot(data = . %>% filter(Plant.species == "Rubus"),
               aes(x=Plant.species, y=Rubus.QC.call.rate, color=Plant.species)) +
  geom_boxplot(data = . %>% filter(Plant.species == "Mānuka"),
               aes(x=Plant.species, y=Mānuka.QC.call.rate, color=Plant.species)) +
  geom_boxplot(data = . %>% filter(Fish.species == "Snapper"),
               aes(x=Fish.species, y=Snapper.QC.call.rate, color=Fish.species)) +
  geom_boxplot(data = . %>% filter(Fish.species == "Trevally"),
               aes(x=Fish.species, y=Trevally.QC.call.rate, color=Fish.species)) +
  geom_boxplot(data = . %>% filter(Fish.species == "Seabream"),
               aes(x=Fish.species, y=Snapper.QC.call.rate, color=Fish.species)) +
  geom_boxplot(data = . %>% filter(Fish.species == "Kingfish"),
               aes(x=Fish.species, y=Trevally.QC.call.rate, color=Fish.species)) +
  xlab("Species") + ylab("QC call rate") +
  theme(legend.position = "none", axis.title = element_text(size = 20), axis.text = e
  geom_hline(yintercept=95, size=0.2)
```

```{r call rate boxplots}
table %>% ggplot() +
  geom_boxplot(data = . %>% filter(Plant.species == "Rubus"),
               aes(x=Plant.species, y=Rubus.call.rate, color=Plant.species)) +
  geom_boxplot(data = . %>% filter(Plant.species == "Mānuka"),
```
```

````
              aes(x=Plant.species, y=Mānuka.call.rate, color=Plant.species)) +
  geom_boxplot(data = . %>% filter(Fish.species == "Snapper"),
              aes(x=Fish.species, y=Snapper.call.rate, color=Fish.species)) +
  geom_boxplot(data = . %>% filter(Fish.species == "Trevally"),
              aes(x=Fish.species, y=Trevally.call.rate, color=Fish.species)) +
  geom_boxplot(data = . %>% filter(Fish.species == "Seabream"),
              aes(x=Fish.species, y=Snapper.call.rate, color=Fish.species)) +
  geom_boxplot(data = . %>% filter(Fish.species == "Kingfish"),
              aes(x=Fish.species, y=Trevally.call.rate, color=Fish.species)) +
  xlab("Species") + ylab("Call rate") +
  theme(legend.position = "none", axis.title = element_text(size = 20), axis.text = e
```

```{r boxplots by species and pooling}
# DQC
p3 <- table %>% select(Plant.species, Fish.species, Rubus.DQC, Mānuka.DQC) %>%
  filter(Plant.species != "none") %>%
  mutate(Plant_pooled = ifelse(Fish.species != "none", "pooled", "not_pooled")) %>%
  mutate(Plant.DQC = ifelse(Plant.species == "Rubus", Rubus.DQC, Mānuka.DQC)) %>%
  ggplot() +
  geom_boxplot(aes(x=Plant.species, y=Plant.DQC, color=Plant_pooled)) +
  xlab("Plant species") + ylab("DQC") +
  theme(legend.position = "none", axis.title = element_text(size = 20), axis.text = e
  geom_hline(yintercept=0.82, size=0.2)

p4 <- table %>% select(Plant.species, Fish.species, Snapper.DQC, Trevally.DQC) %>%
  filter(Fish.species == "Snapper" | Fish.species == "Trevally") %>%
  mutate(Fish_pooled = ifelse(Plant.species != "none", "pooled", "not_pooled")) %>%
  mutate(Fish.DQC = ifelse(Fish.species == "Snapper", Snapper.DQC, Trevally.DQC)) %>%
  ggplot() +
  geom_boxplot(aes(x=Fish.species, y=Fish.DQC, color=Fish_pooled)) +
  xlab("Fish species") + ylab("DQC") +
  guides(color=guide_legend(title="DNA pooling")) +
  theme(axis.title.y=element_blank(), axis.text.y=element_blank(), axis.ticks.y=eleme
        axis.title.x = element_text(size = 20), axis.text.x = element_text(size = 16)
        legend.title = element_text(size=20), legend.text = element_text(size=16)) +
  geom_hline(yintercept=0.82, size=0.2)

grid.arrange(p3, p4, nrow = 1, widths = c(4.5, 6))

# QC CR
p5 <- table %>% select(Plant.species, Fish.species, Rubus.QC.call.rate, Mānuka.QC.cal
  filter(Plant.species != "none") %>%
  mutate(Plant_pooled = ifelse(Fish.species != "none", "pooled", "not_pooled")) %>%
  mutate(Plant.QC.call_rate = ifelse(Plant.species == "Rubus", Rubus.QC.call.rate, Mā
  ggplot() +
  geom_boxplot(aes(x=Plant.species, y=Plant.QC.call_rate, color=Plant_pooled)) +
  xlab("Plant species") + ylab("QC call rate") +
  theme(legend.position = "none", axis.title = element_text(size = 20), axis.text = e
  geom_hline(yintercept=95, size=0.2)

p6 <- table %>% select(Plant.species, Fish.species, Snapper.QC.call.rate, Trevally.QC
  filter(Fish.species == "Snapper" | Fish.species == "Trevally") %>%
  mutate(Fish_pooled = ifelse(Plant.species != "none", "pooled", "not_pooled")) %>%
  mutate(Fish.QC.call_rate = ifelse(Fish.species == "Snapper", Snapper.QC.call.rate,
  ggplot() +
  geom_boxplot(aes(x=Fish.species, y=Fish.QC.call_rate, color=Fish_pooled)) +
  xlab("Fish species") + ylab("QC call rate") +
````

````
  guides(color=guide_legend(title="DNA pooling")) +
  theme(axis.title.y=element_blank(), axis.text.y=element_blank(), axis.ticks.y=eleme
        axis.title.x = element_text(size = 20), axis.text.x = element_text(size = 16)
        legend.title = element_text(size=20), legend.text = element_text(size=16)) +
  geom_hline(yintercept=95, size=0.2)

grid.arrange(p5, p6, nrow = 1, widths = c(4.5, 6))


# Call rate
p7 <- table %>% select(Plant.species, Fish.species, Rubus.call.rate, Mānuka.call.rate
  filter(Plant.species != "none") %>%
  mutate(Plant_pooled = ifelse(Fish.species != "none", "pooled", "not_pooled")) %>%
  mutate(Plant.call.rate = ifelse(Plant.species == "Rubus", Rubus.call.rate, Mānuka.c
  ggplot() +
  geom_boxplot(aes(x=Plant.species, y=Plant.call.rate, color=Plant_pooled)) +
  xlab("Plant species") + ylab("Call rate") +
  theme(legend.position = "none", axis.title = element_text(size = 20), axis.text = e

p8 <- table %>% select(Plant.species, Fish.species, Snapper.call.rate, Trevally.call.
  filter(Fish.species == "Snapper" | Fish.species == "Trevally") %>%
  mutate(Fish_pooled = ifelse(Plant.species != "none", "pooled", "not_pooled")) %>%
  mutate(Fish.call.rate = ifelse(Fish.species == "Snapper", Snapper.call.rate, Treval
  ggplot() +
  geom_boxplot(aes(x=Fish.species, y=Fish.call.rate, color=Fish_pooled)) +
  xlab("Fish species") + ylab("Call rate") +
  guides(color=guide_legend(title="DNA pooling")) +
  theme(axis.title.y=element_blank(), axis.text.y=element_blank(), axis.ticks.y=eleme
        axis.title.x = element_text(size = 20), axis.text.x = element_text(size = 16)
        legend.title = element_text(size=20), legend.text = element_text(size=16))

grid.arrange(p7, p8, nrow = 1, widths = c(4.5, 6))
```

## Perform a Unequal Variance (Indpendent) T-test (Welch T-test) for two sample sets
```{r Welch t-test}
# DQC plant
table_DQC_plants <-
  table %>% select(Plant.species, Fish.species, Rubus.DQC, Mānuka.DQC) %>%
  filter(Plant.species != "none") %>%
  mutate(Plant_pooled = ifelse(Fish.species != "none", "pooled", "not_pooled")) %>%
  mutate(Plant.DQC = ifelse(Plant.species == "Rubus", Rubus.DQC, Mānuka.DQC)) %>%
  select(Plant_pooled, Plant.DQC)
t.test(table_DQC_plants$Plant.DQC ~ table_DQC_plants$Plant_pooled)

# DQC fish
table_DQC_fish <-
  table %>% select(Plant.species, Fish.species, Snapper.DQC, Trevally.DQC) %>%
  filter(Fish.species == "Snapper" | Fish.species == "Trevally") %>%
  mutate(Fish_pooled = ifelse(Plant.species != "none", "pooled", "not_pooled")) %>%
  mutate(Fish.DQC = ifelse(Fish.species == "Snapper", Snapper.DQC, Trevally.DQC)) %>%
  select(Fish_pooled, Fish.DQC)
t.test(table_DQC_fish$Fish.DQC ~ table_DQC_fish$Fish_pooled)

# QC CR plant
table_QCCR_plants <-
  table %>% select(Plant.species, Fish.species, Rubus.QC.call.rate, Mānuka.QC.call.ra
  filter(Plant.species != "none") %>%
````

````
  mutate(Plant_pooled = ifelse(Fish.species != "none", "pooled", "not_pooled")) %>%
  mutate(Plant.QC.call_rate = ifelse(Plant.species == "Rubus", Rubus.QC.call.rate, Mā
  select(Plant_pooled, Plant.QC.call_rate)
t.test(table_QCCR_plants$Plant.QC.call_rate ~ table_QCCR_plants$Plant_pooled)

#QC Cr fish
table_QCCR_fish <-
  table %>% select(Plant.species, Fish.species, Snapper.QC.call.rate, Trevally.QC.cal
  filter(Fish.species == "Snapper" | Fish.species == "Trevally") %>%
  mutate(Fish_pooled = ifelse(Plant.species != "none", "pooled", "not_pooled")) %>%
  mutate(Fish.QC.call_rate = ifelse(Fish.species == "Snapper", Snapper.QC.call.rate,
  select(Fish_pooled, Fish.QC.call_rate)
t.test(table_QCCR_fish$Fish.QC.call_rate ~ table_QCCR_fish$Fish_pooled)

# Call rate plant
table_CR_plants <-
  table %>% select(Plant.species, Fish.species, Rubus.call.rate, Mānuka.call.rate) %>
  filter(Plant.species != "none") %>%
  mutate(Plant_pooled = ifelse(Fish.species != "none", "pooled", "not_pooled")) %>%
  mutate(Plant.call.rate = ifelse(Plant.species == "Rubus", Rubus.call.rate, Mānuka.c
  select(Plant_pooled, Plant.call.rate)
t.test(table_CR_plants$Plant.call.rate ~ table_CR_plants$Plant_pooled)

#QC Cr fish
table_CR_fish <-
  table %>% select(Plant.species, Fish.species, Snapper.call.rate, Trevally.call.rate
  filter(Fish.species == "Snapper" | Fish.species == "Trevally") %>%
  mutate(Fish_pooled = ifelse(Plant.species != "none", "pooled", "not_pooled")) %>%
  mutate(Fish.call.rate = ifelse(Fish.species == "Snapper", Snapper.call.rate, Treval
  select(Fish_pooled, Fish.call.rate)
t.test(table_CR_fish$Fish.call.rate ~ table_CR_fish$Fish_pooled)
```
at alpha = 0.05, all differences are significant except for QCCR for fish

```{r scatterplots plant vs fish from same reaction}
# DQC
table %>% select(Plant.species, Fish.species, Rubus.DQC, Mānuka.DQC, Snapper.DQC, Tre
  filter(Plant.species != "none") %>%
  filter(Fish.species != "none") %>%
  mutate(Plant.DQC = ifelse(Plant.species == "Rubus", Rubus.DQC, Mānuka.DQC)) %>%
  mutate(Fish.DQC = ifelse(Fish.species == "Snapper", Snapper.DQC, Trevally.DQC)) %>%
  ggplot() +
  geom_point(aes(x=Plant.DQC, y=Fish.DQC)) +
  theme(axis.title = element_text(size = 20), axis.text = element_text(size = 16)) +
  geom_hline(yintercept=0.82, color="red", size=0.2) +
  geom_vline(xintercept=0.82, color="red", size=0.2)

# QCCR
table %>% select(Plant.species, Fish.species, Rubus.QC.call.rate, Mānuka.QC.call.rate
  filter(Plant.species != "none") %>%
  filter(Fish.species != "none") %>%
  mutate(Plant.QC.call.rate = ifelse(Plant.species == "Rubus", Rubus.QC.call.rate, Mā
  mutate(Fish.QC.call.rate = ifelse(Fish.species == "Snapper", Snapper.QC.call.rate,
  ggplot() +
  geom_point(aes(x=Plant.QC.call.rate, y=Fish.QC.call.rate)) +
  theme(axis.title = element_text(size = 20), axis.text = element_text(size = 16)) +
  geom_hline(yintercept=95, color="red", size=0.2) +
  geom_vline(xintercept=95, color="red", size=0.2)
````

```
# Call rate
table %>% select(Plant.species, Fish.species, Rubus.call.rate, Mānuka.call.rate, Snap
  filter(Plant.species != "none") %>%
  filter(Fish.species != "none") %>%
  mutate(Plant.call.rate = ifelse(Plant.species == "Rubus", Rubus.call.rate, Mānuka.c
  mutate(Fish.call.rate = ifelse(Fish.species == "Snapper", Snapper.call.rate, Treval
  ggplot() +
  geom_point(aes(x=Plant.call.rate, y=Fish.call.rate)) +
  theme(axis.title = element_text(size = 20), axis.text = element_text(size = 16))
```

```{r correlation QCCR with call rate}
Rubus <- table %>% select(Rubus.QC.call.rate, Rubus.call.rate) %>%
  na.omit() %>%
  filter(Rubus.QC.call.rate >= 95) %>%
  mutate(Species = "Rubus") %>%
  rename(QC.call.rate = Rubus.QC.call.rate) %>%
  rename(Call.rate = Rubus.call.rate)
Mānuka <- table %>% select(Mānuka.QC.call.rate, Mānuka.call.rate) %>%
  na.omit() %>%
  mutate(Species = "Mānuka") %>%
  rename(QC.call.rate = Mānuka.QC.call.rate) %>%
  rename(Call.rate = Mānuka.call.rate)
Snapper <- table %>% select(Snapper.QC.call.rate, Snapper.call.rate) %>%
  na.omit() %>%
  mutate(Species = "Snapper") %>%
  rename(QC.call.rate = Snapper.QC.call.rate) %>%
  rename(Call.rate = Snapper.call.rate)
Trevally <- table %>% select(Trevally.QC.call.rate, Trevally.call.rate) %>%
  na.omit() %>%
  mutate(Species = "Trevally") %>%
  rename(QC.call.rate = Trevally.QC.call.rate) %>%
  rename(Call.rate = Trevally.call.rate)

rbind(Rubus, Mānuka, Snapper, Trevally) %>%
  ggplot() +
  geom_point(aes(x=QC.call.rate, y=Call.rate, color=Species), alpha=0.3, shape=1) +
  geom_smooth(aes(x=QC.call.rate, y=Call.rate, color=Species), method = "loess", size
  theme(axis.title = element_text(size = 20), axis.text = element_text(size = 16))
```

```{r DQC vs QC call rate by pooling}
# Plants
table %>% select(Plant.species, Fish.species, Rubus.DQC, Mānuka.DQC, Rubus.QC.call.ra
  filter(Plant.species != "none") %>%
  mutate(Plant_pooled = ifelse(Fish.species != "none", "pooled", "not_pooled")) %>%
  mutate(Plant.DQC = ifelse(Plant.species == "Rubus", Rubus.DQC, Mānuka.DQC)) %>%
  mutate(Plant.QC.call_rate = ifelse(Plant.species == "Rubus", Rubus.QC.call.rate, Mā
  ggplot() +
  geom_point(aes(x=Plant.DQC, y=Plant.QC.call_rate, colour = Plant_pooled), alpha = 0
  xlim(0.80, 1) +
  guides(color=guide_legend(title="DNA pooling")) +
  theme(axis.title = element_text(size = 20), axis.text = element_text(size = 16),
        legend.title = element_text(size=20), legend.text = element_text(size=16)) +
  geom_hline(yintercept=95, color="red", size=0.2)

# Fishes
```

```
table %>% select(Plant.species, Fish.species, Snapper.DQC, Trevally.DQC, Snapper.QC.c
  filter(Fish.species == "Snapper" | Fish.species == "Trevally") %>%
  mutate(Fish_pooled = ifelse(Plant.species != "none", "pooled", "not_pooled")) %>%
  mutate(Fish.QC.call_rate = ifelse(Fish.species == "Snapper", Snapper.QC.call.rate,
  mutate(Fish.DQC = ifelse(Fish.species == "Snapper", Snapper.DQC, Trevally.DQC)) %>%
  ggplot() +
  geom_point(aes(x=Fish.DQC, y=Fish.QC.call_rate, colour = Fish_pooled), alpha = 0.3)
  xlim(0.80, 1) +
  guides(color=guide_legend(title="DNA pooling")) +
  theme(axis.title = element_text(size = 20), axis.text = element_text(size = 16),
        legend.title = element_text(size=20), legend.text = element_text(size=16)) +
  geom_hline(yintercept=95, color="red", size=0.2)
```
```
```
