# Supplementary material for: A multiplexed plant–animal SNP array for selective breeding and species conservation applications
Source: G3 (Bethesda). 2023 Aug 11;13(10):jkad170. doi: 10.1093/g3journal/jkad170 (PMC10542201; doi:10.1093/g3journal/jkad170)
Supplement: jkad170_Supplementary_Data [file jkad170_supplementary_data.zip › Supplemental_Material_Legends_G3-2023-404167.docx]

# Supplemental Material legends

**Figure S1. Cluster plot evaluation in *Rubus*.** Cluster plots for two *Rubus* single nucleotide polymorphism (SNP) markers classified as *PolyHighResolution*. Highlighted in pink are samples with an allele_deviation_mean > 0.85.

**Figure S2. Cluster plot evaluation for Japanese red seabream samples.** Cluster plots for snapper single nucleotide polymorphism (SNP) markers classified as *PolyHighResolution* (A), *NoMinorHom* (B) and *OffTargetVariant* (C and D). Highlighted in pink are seabream samples, while the others are snapper samples.

**Figure S3. Variant calling filtering steps for raspberry (*Rubus* subgenus *Idaeobatus*).** The diagram shows the filtering parameters above the arrow line, and the number of single nucleotide polymorphisms (SNPs) left at each step below the arrow line. Each raspberry family is represented with a different color. Q = RMS mapping quality; DP = depth; MAF = minor allele frequency; LGs = Linkage Groups.

**Table S1. Samples screened with the multi-species plant animal single nucleotide polymorphism (SNP) array.** For each sample, the genotyping batch, the plant and animal species pooled and the relative DQC, QC call rate and call rate values are reported.

**Table S2. Raspberry (*Rubus* subgenus *Idaeobatus*) F_1_ populations sequenced via genotyping-by-sequencing and used for variant calling.**

**Table S3. Classes of mānuka single nucleotide polymorphisms (SNPs) identified based on their minor allele frequency (MAF) values in each or a combination of gene pools.**

**Table S4. Details of the single nucleotide polymorphism (SNP) markers included in the multi-species 60K SNP array.** For each SNP the position on the reference genome and the flanking sequences are reported.

**Table S5. Number of single nucleotide polymorphism (SNP) markers by species and Axiom classification.**

**Table S6. Mānuka samples used for single nucleotide polymorphism (SNP) validation.** For each sample, the region of origin is reported. A table exhibiting the number of samples per provenance is also reported.

**Table S7. Trevally samples used for single nucleotide polymorphism (SNP) validation.** For each sample, the country of harvesting is reported. A table exhibiting the number of samples per country of origin is also reported.

**Table S8. Blackberry (*Rubus* subgenus *Rubus*) accessions re-sequenced for variant calling.** For each genotype, the repository of origin, the ploidy and the total paired-end reads are reported.

**File S1. Full scripts used for evaluation of the genotypic data and SNP validation.**
