# Supplementary figures and images for: A multiplexed plant–animal SNP array for selective breeding and species conservation applications
Source: G3 (Bethesda). 2023 Aug 11;13(10):jkad170. doi: 10.1093/g3journal/jkad170 (PMC10542201; doi:10.1093/g3journal/jkad170)

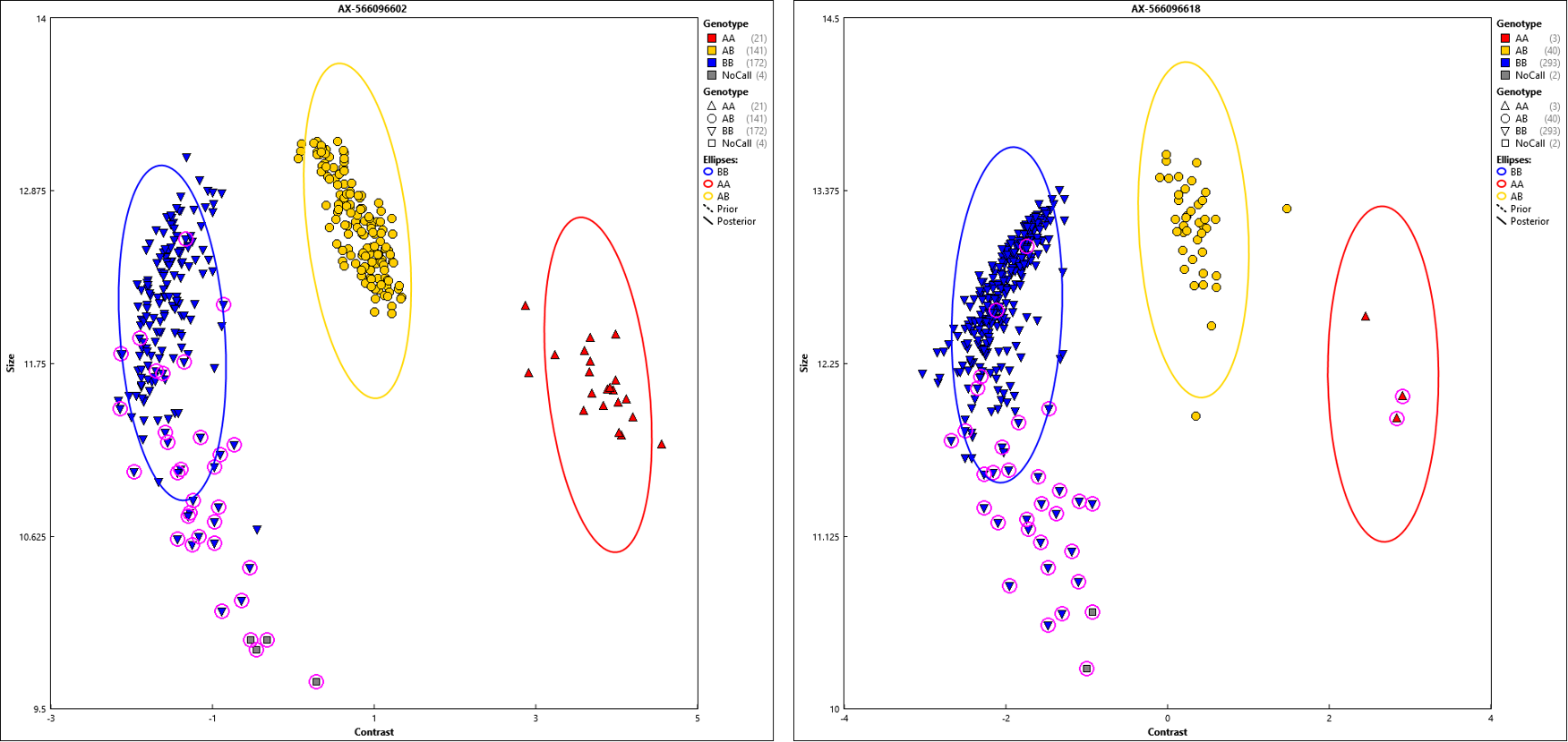

Supplement: jkad170_Supplementary_Data [file jkad170_supplementary_data.zip › Figure_S1_G3-2023-404167.tif]

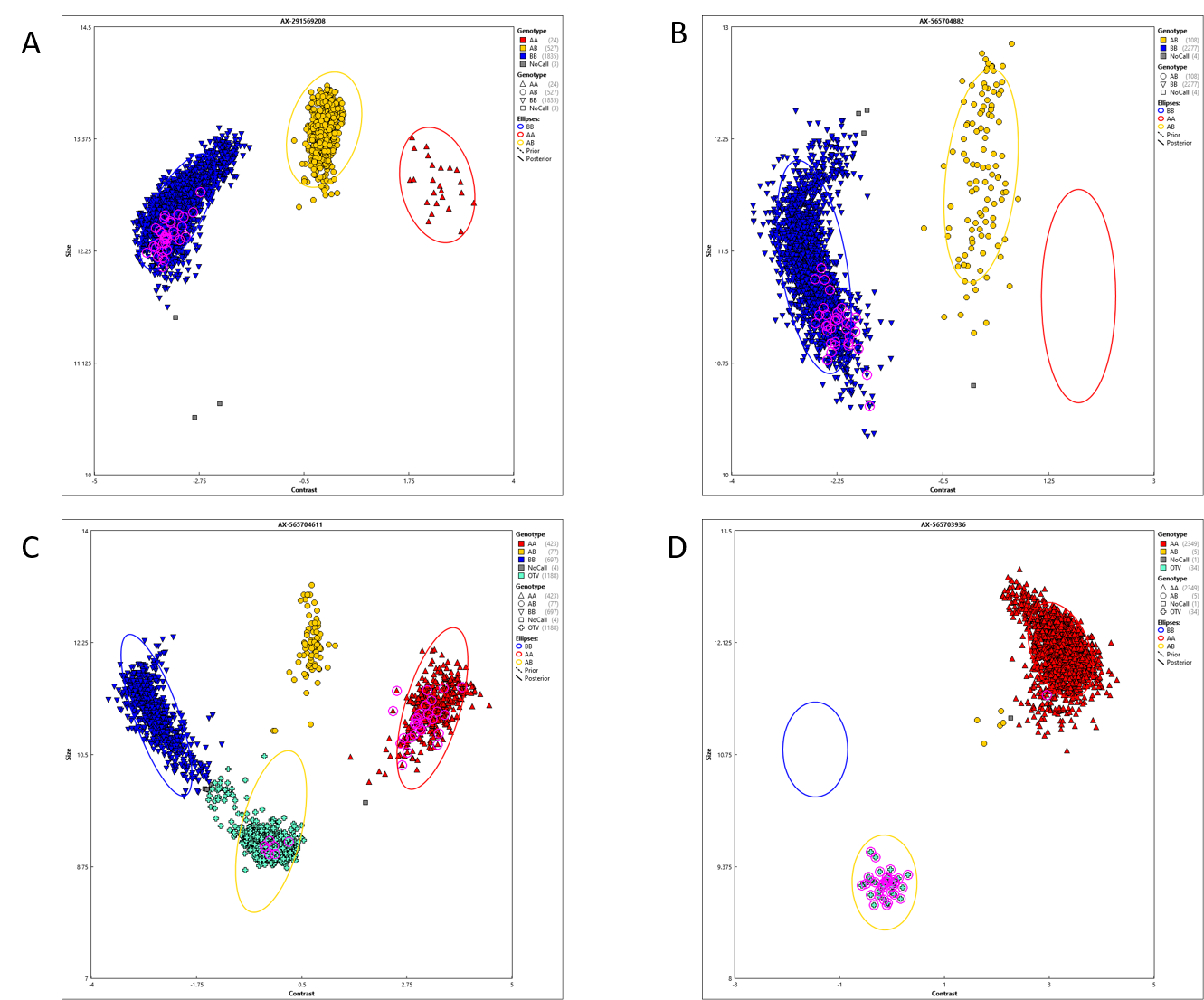

Supplement: jkad170_Supplementary_Data [file jkad170_supplementary_data.zip › Figure_S2_G3-2023-404167.tif]

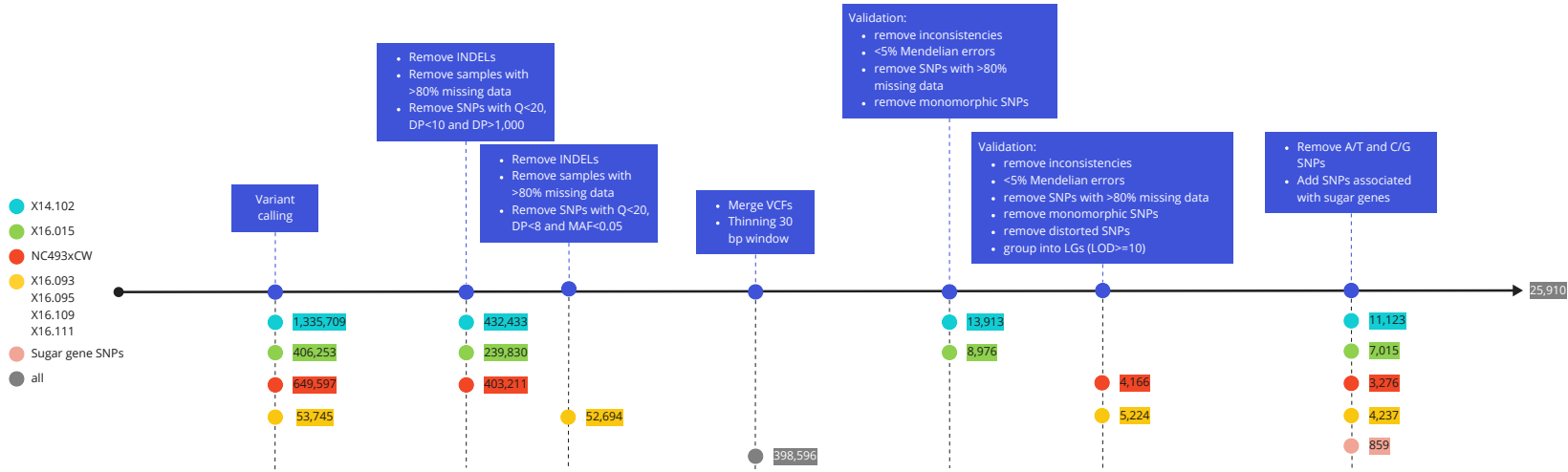

Supplement: jkad170_Supplementary_Data [file jkad170_supplementary_data.zip › Figure_S3_G3-2023-404167.pdf]
